# Metabolic score for visceral fat as a predictor of new-onset cardiovascular disease in patients with metabolic syndrome: a validation study in two prospective cohorts (UK Biobank and CHARLS)

**DOI:** 10.1186/s12872-026-06299-2

**Published:** 2026-08-01

**Authors:** Zhen Tan, Yijun Liu, Lei Liu, Mao Ye, Xinrui Xue, Shuang Li, Xiaoping Li, Hongqiang Ren

**Affiliations:** 1 Department of Cardiology, Suining Central Hospital, No.27 of Dongping North Road, Hedong New District, Suining, Sichuan China; 2 Department of Cardiology, Sichuan Provincial People’s Hospital, University of Electronic Science and Technology of China, Chengdu, Sichuan China

**Keywords:** Metabolism score for visceral fat, New-onset cardiovascular disease, Metabolic syndrome, UK Biobank, China health and retirement longitudinal study

## Abstract

**Background:**

Metabolic syndrome (MetS) substantially increases cardiovascular disease (CVD) risk. The metabolism score for visceral fat (METS-VF) is a novel assessment tool that may substitute visceral adipose tissue measurement. This study investigated the association between METS-VF and new-onset CVD in MetS patients.

**Methods:**

The data from two prospective cohorts: UK Biobank (UKB) and the China Health and Retirement Longitudinal Study (CHARLS). Multivariate Cox regression and restricted cubic spline (RCS) analyses assessed the association between METS-VF and new-onset CVD. The interaction between METS-VF and the CVD polygenic risk score (PRS) was analyzed in the UKB to assess genetic contributions. Receiver operating characteristic (ROC) curves, net reclassification improvement (NRI), and integrated discrimination improvement (IDI) were used to evaluate the discriminative capability of METS-VF for new-onset CVD. Subgroup analyses were performed to confirm the robustness of the results.

**Results:**

A total of 101,292 individuals from the UK Biobank and 1,680 individuals from CHARLS were included. The median follow-up periods were 14.6 years in UKB and 5.0 years in CHARLS. High METS-VF was significantly associated with an increased risk of new-onset CVD in both UKB (HR = 1.63, 95% CI: 1.56–1.71) and CHARLS (HR = 2.11, 95% CI: 1.52–2.94) compared with low METS-VF. Individuals with the highest METS-VF and high genetic risk exhibited the highest risk of new-onset CVD (HR = 2.36, 95% CI: 2.14–2.61).

**Conclusions:**

METS-VF is a valuable indicator for predicting new-onset CVD in individuals with MetS, providing new insights into the prevention and management of CVD in high-risk populations.

**Trial registration:**

Not applicable.

**Supplementary Information:**

The online version contains supplementary material available at https://doi.org/10.1186/s12872-026-06299-2.

## Background

Metabolic syndrome (MetS) prevalence has risen globally over recent decades due to population ageing, physical inactivity, and poor diet—making it a leading public health priority [1]. MetS is a clinical condition characterized by the concurrent presence of central adiposity, elevated fasting glucose, dyslipidaemia and elevated blood pressure, and it is strongly associated with an increased risk of new-onset cardiovascular disease (CVD), particularly in individuals with visceral fat-mediated metabolic impairment [2, 3]. Epidemiological data show MetS affects 20–30% of adults in developed countries [4], while in China, the prevalence reaches 31% (over 450 million people) [5]. This upward trend, tied to rising obesity and sedentary behaviours, places a growing burden on global healthcare systems [6].

The impact of MetS extends far beyond its immediate symptoms, particularly concerning CVD, which are among the leading causes of morbidity and mortality in individuals with MetS [7]. Compared with the general population, individuals with MetS exhibit a significantly higher risk of CVD, CVD-related death, and all-cause mortality [8]. Additionally, MetS is associated with an increased risk of developing type 2 diabetes and several cancers, including breast, endometrial, prostate, pancreatic, hepatobiliary, and colorectal cancers [9–13]. Given these significant risks, understanding the underlying mechanisms linking MetS to CVD is crucial for the development of effective preventive and therapeutic strategies.

Visceral adiposity is a crucial pathophysiological driver of both MetS and its cardiovascular complications [14]. As a primary component of central obesity, Visceral adiposity functions not only as a depot for lipid accumulation but also as a dynamic endocrine and paracrine organ that secretes hormones, inflammatory mediators, and metabolic regulators, such as tumour necrosis factor-alpha (TNF-α), interleukin-6 (IL-6), and adiponectin [15–17]. These substances disrupt both metabolic and cardiovascular homeostasis, leading to insulin resistance, dyslipidaemia, and endothelial dysfunction [18–20]. Therefore, quantifying the metabolic function of visceral fat could provide critical insights into CVD risk stratification in individuals with MetS.

The metabolic score for visceral fat (METS-VF), which is derived from the metabolic score for insulin resistance (METS-IR), waist-to-height ratio (WHtR), age and sex, has been identified as a novel biomarker [21]. It integrates multiple metabolic parameters linked to visceral fat function, including lipid metabolism, glucose metabolism and adipokine secretion [22]. METS-VF offers a more comprehensive and accurate assessment compared to traditional measures of adiposity, such as body mass index (BMI), waist circumference (WC), and visceral adiposity index (VAI) [23]. Previous studies have demonstrated that METS-VF is independently associated with coronary artery calcification, non-alcoholic fatty liver disease, and diabetes [24–26].

However, the relationship between METS-VF and new-onset CVD in individuals with MetS remains unclear. To identify high-risk individuals with MetS and improve CVD prevention strategies, this study aims to investigate the association between METS-VF and new-onset CVD in two large cohorts of MetS patients.

## Methods

### Study design and population

This study was based on two large-scale prospective cohort studies: the UK Biobank (UKB) and the China Health and Retirement Longitudinal Study (CHARLS). The UKB is a population-based biomedical database covering more than 500,000 participants aged 40–69 years from the United Kingdom, which collects detailed biological, clinical, lifestyle, and genetic data of participants [27]. The UKB has obtained ethical approval from the North West Multi-centre Research Ethics Committee (Research Tissue Bank approval), and this study was conducted under the UKB research license (Application ID: 106027). CHARLS is a national longitudinal cohort study in China, which aims to study the health and aging problems of Chinese residents aged 45 years and above [28]. The baseline survey was conducted in 2011–2012, with follow-up surveys every 2–3 years, and five rounds of surveys have been completed to date. The CHARLS protocol was approved by the Ethical Review Committee of Suining Central Hospital (Approval number: KYLLMC20250034).

The diagnosis of MetS was based on the presence of at least three of the following five criteria [29]: (1) Obesity: waist circumference ≥ 102 cm in men and ≥ 88 cm in women; (2) Elevated triglycerides: ≥ 150 mg/dL (1.7 mmol/L) or use of lipid-lowering drugs; (3) Elevated blood pressure: systolic blood pressure (SBP) ≥ 130 mmHg or diastolic blood pressure (DBP) ≥ 85 mmHg, or a confirmed diagnosis of hypertension or use of antihypertensive drugs; (4) Elevated fasting blood glucose (FBG): ≥ 100 mg/dL (5.6 mmol/L), or a confirmed diagnosis of diabetes or use of antidiabetic drugs; (5) Reduced high-density lipoprotein cholesterol (HDL-C): < 40 mg/dL (1.0 mmol/L) in men and < 50 mg/dL (1.3 mmol/L) in women, or use of lipid-lowering drugs.

Exclusion criteria included: (1) participants with a history of angina, myocardial infarction (MI), ischemic stroke (ISS), hemorrhagic stroke, atrial fibrillation (AF), or heart failure (HF) before enrollment; (2) participants with missing data on WC, weight, height, triglycerides (TG), HDL-C, FBG, and fasting plasma glucose (FPG) at baseline; (3) participants who were unable or unwilling to provide informed consent; and (4) participants lost to follow-up during the study period. The detailed screening process of the study population is shown in Supplementary Figure S1.

### Assessment covariates and METS-VF

At baseline, standardized computerized questionnaires were used to collect demographic and lifestyle information from all participants, including age, gender, race (UKB only), education level, smoking status (never, former, current), alcohol consumption status (never, former, current), physical activity frequency (never, < 3 times per week, or ≥ 3 times per week; UKB only), and household income. Clinical information such as medical history (hypertension, diabetes) and medication use (aspirin, antihypertensive drugs, lipid-lowering drugs) was also collected. Blood samples were collected after an overnight fast of at least 8 h to measure FPG, TG, HDL-C, low-density lipoprotein cholesterol (LDL-C), and other metabolic indicators. Body mass index (BMI) was calculated as weight (kg) divided by height squared (m²), and waist-to-height ratio (WHtR) was calculated as waist circumference (cm) divided by height (cm).

The relevant formulae are as follows [25, 30]:

$$\begin{aligned}METS-VF&=4.466+0.011\left[\left(Ln\left(METS-IR\right)\right)\right]^3\\&+3.239\left[\left(Ln\left(WHTR\right)\right)^3\right]+0.319\left(gender\right)\\&+0.594\left(Ln\left(age\right)\right)\left(male=1,female=2\right)\end{aligned}$$

$$\begin{aligned}METS-IR&=Ln\lbrack2\times(FPG(mg/dL)+TG(mg/dL))\rbrack\\&\times BMI(kg/m^2)/Ln\lbrack HDL-C(mg/dL)\rbrack\end{aligned}$$

$$BMI=Weight(kg)/Height^2(m^2)$$

$$\begin{aligned}Triglyceride&-Glucoseindex(TyG)\\&=Ln\lbrack TG(mg/dl)\\&\times FBG(mg/dl)/2\rbrack\end{aligned}$$

$$\begin{aligned}VAI&=WC(cm)/(39.68+(1.88\times BMI)\\&\times(TG(mmol/L)/1.03)\\&\times(1.31/HDL-C(mmol/L))(male)\end{aligned}$$

$$\begin{aligned}VAI&=WC\left(cm\right)/\left(36.58+\left(1.89\times BMI\right)\right)\\&\times\left(TG\left(mmol/L\right)/0.81\right)\\&\times\left(1.52/HDL-C\left(mmol/L\right)\right)\left(female\right)\end{aligned}$$

All participants in the UKB and CHARLS cohorts were divided into four groups (Q1, Q2, Q3, Q4) according to the 25th, 50th, and 75th percentiles of METS-VF levels, with Q1 (the lowest quartile) as the reference group for subsequent statistical analysis.

### Outcome definition and follow-up

The primary outcome of this study was new-onset CVD during the follow-up period. In the UKB cohort, CVD was defined according to the International Statistical Classification of Diseases, 10th Revision (ICD-10), including ischemic heart disease (IHD): angina (I20), myocardial infarction (MI; I21-I23); stroke: ischemic stroke (ISS; I63), hemorrhagic stroke (I60-I62); atrial fibrillation (AF; I48); and heart failure (HF; I50) [31]. The follow-up period in the UKB cohort was from the date of the first re-examination to the date of CVD diagnosis, study withdrawal (due to death or loss to follow-up), or the end of the latest follow-up (December 19, 2022), whichever came first.

In the CHARLS cohort, new-onset CVD was defined as self-reported, physician-diagnosed heart disease or stroke during the follow-up period [28]. Heart disease included MI, coronary heart disease, angina, HF, and other heart diseases. The follow-up period was from Wave 1 (2011–2012) to Wave 5 (2020–2021), and the outcome was the first occurrence of heart disease or stroke during the this period. The follow-up cut-off date was the date of CVD diagnosis or the end of Wave 5 (2019–2020).

### Definition of polygenic risk score

The standardized polygenic risk score (PRS) for CVD, coronary artery disease (CAD), AF, and ISS from the UKB cohort was used in this study. PRS was calculated by summing the product of the effect size of each genetic variant and the allele dosage, based on genome-wide association study (GWAS) summary statistics and Bayesian meta-analysis [32]. According to the PRS values, participants were divided into three genetic risk groups: low genetic risk (1st quintile), intermediate genetic risk (2nd-4th quintiles), and high genetic risk (5th quintile). For the analysis of IHD, angina, and MI, CAD-PRS was used. For other outcomes, the corresponding standardized PRS was used.

### Statistical analysis

All statistical analyses were performed using R software (version 4.3.0; Vienna, Austria). Continuous variables were expressed as mean ± standard deviation (SD) if normally distributed, and as median (interquartile range [IQR]) if non-normally distributed. Categorical variables were expressed as frequency and percentage (n, %). The Wilcoxon rank-sum test was used to compare non-normally distributed continuous variables between groups, and the chi-square test was used for categorical variables. Missing covariate values were addressed using Multiple Imputation by Chained Equations (MICE), and 5 imputed datasets were created. Among all covariates, the maximum missing rate was 7.2% (UKB cohort ) and 2.3% (CHARLS cohort ) and the average missing rate was 1.86% (UKB cohort ) and 0.39% (CHARLS cohort ) (Table S1). We assessed the stability of imputation using Monte Carlo error analysis; all errors were low, confirming reliable imputation results.

Multivariate Cox proportional hazards regression models were used to analyze the association between METS-VF and new-onset CVD, and three models were constructed for adjustment in each cohort: Model 1: Unadjusted model; Model 2: Adjusted for basic confounding factors (gender, age, education, race, smoking, alcohol consumption, physical activity); Model 3: Fully adjusted Cox model based on Model 2, the model further adjusted for clinical covariates (hypertension, diabetes, LDL-C, uric acid, HbA1c). Hazard ratios (HRs) and 95% confidence intervals (CIs) were used to quantify the association between METS-VF and CVD risk. Schoenfeld residual test was applied to verify the proportional hazards assumption for all Cox models, and no violation of the assumption was observed (all *P* > 0.05).

Pooled effect sizes across the two cohorts were calculated using the DerSimonian–Laird random-effects model. Statistical heterogeneity between studies was assessed using the I² statistic and Cochran’s Q test. A predefined threshold of I² ≥ 50% was used to indicate moderate-to-high heterogeneity, for which the random-effects model was applied; otherwise, the fixed-effects model was adopted.

RCS analyses were used to explore the dose-response correlation between METS-VF and new-onset CVD, and the inflection point was calculated if a non-linear correlation was found. Stratified analysis by genetic risk group was performed to explore the joint effect of METS-VF and PRS on CVD risk. In each genetic risk group, participants were divided into four subgroups according to METS-VF quartiles, and the fully adjusted Cox model was used to analyze the association between METS-VF and CVD risk.

ROC curves were plotted to evaluate the predictive ability of METS-VF and traditional indices (METS-IR, BMI, TyG, and VAI) for 3-year, 5-year, and 8-year new-onset CVD, and the area under the ROC curve (AUC) was calculated. Net reclassification improvement (NRI) and integrated discrimination improvement (IDI).

were used to further evaluate the incremental predictive value of METS-VF compared with traditional indices.

In the subgroup analyses, the effects of age, gender, smoking status, and alcohol consumption status on the association between METS-VF and the risk of new-onset CVD were further examined. To address competing events (all-cause mortality among participants without prior CVD), we used the Fine-Gray proportional subdistribution hazards model to estimate subdistribution hazard ratios (sHRs) and 95% CIs for the association between METS-VF and new-onset CVD [33]. METS-VF was assessed both as a continuous variable (per 1 SD increase) and a categorical variable defined by quartiles, with the Q1 as the reference group. To further verify the robustness of these primary results, we conducted two sensitivity analyses by excluding metabolic variables (hypertension, diabetes, LDL-C, and HbA1c) and excluding those participants taking relevant medications (aspirin, antihypertensive drugs, and lipid-lowering drugs). All primary analyses were performed based on fully adjusted Cox model (model 3). Two-tailed *P* < 0.05 was considered statistically significant.

## Results

### Demographic characteristics of the study participants

A total of 101,292 individuals from the UKB were included in the current study. Among the included individuals, 50,469 (49.83%) were male, and the mean age at baseline was 57.83 ± 7.68 years. Participants were more likely to be overweight, White, drinkers, with hypertension, and have lower levels of education. A total of 1,680 individuals from the CHARLS were included in the current study. Of these, 445 (26.49%) were male, and the median age at baseline was 59 (52–65) years. Participants were more likely to be overweight, drinkers, with hypertension, and high school education or above. Baseline characteristics of the participants across the Q1–Q4 groups in UKB and CHARLS were provided in Supplementary Table S2 and S3.

### Association between METS-VF and new-onset CVD

The median follow-up periods were 14.6 years in UKB and 5.0 years in the CHARLS. New-onset CVD occurred in 21,231 (20.96%) participants from the UKB cohort and 361 (21.49%) participantsfrom the CHARLS cohort. Table 1, Table S4, and Table S5 show that the METS-VF is associated with the risk of new-onset CVD. In UKB, trend tests indicated a significant increase in new-onset CVD risk with increasing METS-VF (*P* < 0.001). The risk of CVD (HR = 1.63, 95% CI: 1.56–1.71), IHD (HR = 1.45, 95% CI: 1.36–1.55), Angina (HR = 1.43, 95% CI: 1.31–1.56), MI (HR = 1.20, 95% CI: 1.08–1.33), stroke (HR = 1.41, 95% CI: 1.25–1.58), ISS (HR = 1.43, 95% CI: 1.26–1.63), hemorrhagic stroke (HR = 1.48, 95% CI: 1.19–1.83), AF (HR = 2.03, 95% CI: 1.89–2.18), and HF (HR = 2.45, 95% CI: 2.22–2.70) were significantly increased in the highest quartile compared to the lowest quartile. When METS-VF was calculated as a continuous variable, each 1-SD increase in METS-VF was associated with 1.24-fold increased risk of new-onset CVD. In CHARLS, trend tests indicated a significant increased in new-onset CVD risk with increasing METS-VF (*P* < 0.001). The risk of CVD (HR = 2.11, 95% CI: 1.52–2.94), heart disease (HR = 2.58, 95% CI: 1.73–3.84), and stroke (HR = 1.63, 95% CI: 1.05–2.53) was significantly increased in the highest quartile compared to the lowest quartile. While each 1-SD increase in METS-VF was associated with 1.87-fold increased risk of new-onset CVD in CHARLS participants.

**Table 1:** Associations of METS-VF with new-onset CVD in UK Biobank and CHARLS

Variable	Cases/Total	*Model 1* HR(95%CI) *P* value	*Model 2* HR(95%CI) *P* value	*Model 3* HR(95%CI) *P* value
UK biobank				
Per 1 SD increase	21,231/101,292	2.24 (2.16–2.33) <0.001	1.89 (1.81–1.96) <0.001	1.65 (1.58–1.71) <0.001
Q1	3709/25,323	Ref	Ref	Ref
Q2	4535/25,323	1.25 (1.19–1.30) <0.001	1.19 (1.13–1.24) <0.001	1.15 (1.09–1.20) <0.001
Q3	5442/25,323	1.53 (1.47–1.60) <0.001	1.36 (1.30–1.42) <0.001	1.27 (1.22–1.33) <0.001
Q4	7545/25,323	2.24 (2.16–2.33) <0.001	1.86 (1.79–1.95) <0.001	1.63 (1.56–1.71) <0.001
P for trend		< 0.001	< 0.001	< 0.001
CHARL				
Per 1 SD increase	361/1680	2.87 (2.02–4.09) <0.001	2.70 (1.91–3.80) <0.001	2.04 (1.35–2.98) <0.001
Q1	65/420	Ref	Ref	Ref
Q2	70/420	1.08 (0.77–1.52) 0.624	1.06 (0.76–1.49) 0.725	1.10 (0.78–1.54) 0.605
Q3	101/420	1.63 (1.19–2.63) 0.002	1.61 (1.17–2.20) 0.003	1.64 (1.19–2.25) 0.002
Q4	125/420	2.11 (1.56–2.85) <0.001	2.07 (1.50–2.87) <0.001	2.11 (1.52–2.94) <0.001
*P for trend*		< 0.001	< 0.001	< 0.001

Per increased in 1-SD of METS-VF was 0.436 and 0.384 for UK Biobank and CHARLS. Model 1: Unadjusted. Model 2: Adjusted for age, gender, race (UK biobank only), education, smoking, alcohol consumption, and physical activity (UK biobank only). Model 3: included all variables from Model 2, and further adjusted for hypertension, diabetes, LDL-C, Ua and HbA1c

*Abbreviations*: *METS-VF* Metabolic score for visceral fat, *CVD* Cardiovascular disease, *HR* Hazard ratios, *CI* Confidence interval, *PRS* Polygenic risk score, *Ref* Reference, *IHD* Ischemic heart disease, *MI* Myocardial infarction, *ISS* Ischemic stroke, *AF* Atrial fibrillation, *LDL-C* Low-density lipoprotein cholesterol, *Ua* Uric acid

Moderate inter-cohort heterogeneity was observed (I² = 56.41%), although the Cochran’s Q test did not reach statistical significance (Q = 2.29, df = 1, *P* = 0.13). According to our predefined criteria, the random-effects model was used for pooled analysis. The meta-analysis demonstrated that higher METS-VF was significantly associated with an increased risk of incident CVD, with a pooled HR of 1.76 (95% CI: 1.40–2.21, *P* < 0.001). This adverse association was consistent across the two independent cohorts. The corresponding forest plot was provided in the supplementary materials (Fig. S2).

**Fig. 1:**
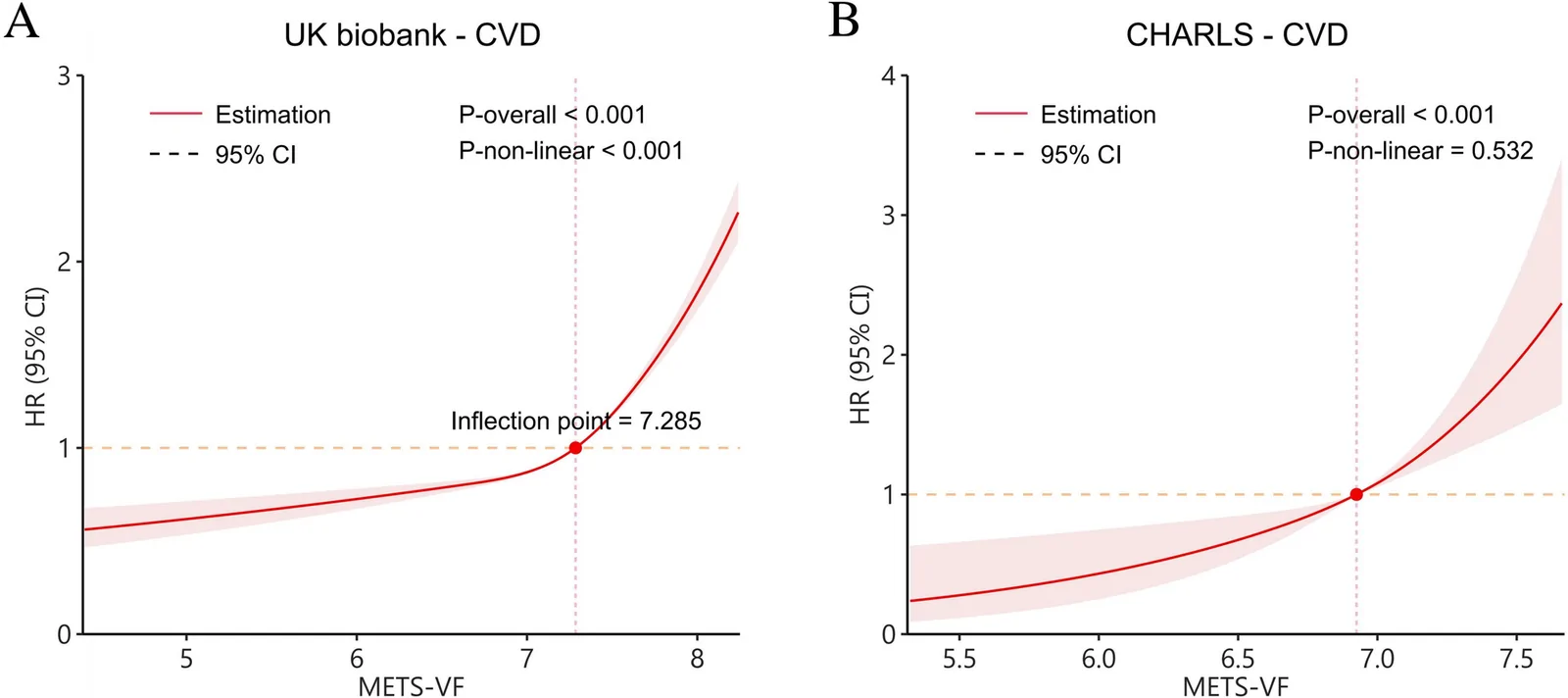
Analysis of restricted cubic spline regression. **A** non-linear relationship between the METS-VF with CVD in UK biobank. **B** A positive linear relationship between the METS-VF with CVD in CHARLS. Adjusted for age, gender, race, education level, smoking status, alcohol consumption status, physical activity, hypertension, diabetes, LDL-C, Ua and HbA1c. Abbreviations: HR: Hazard ratios; CI: Confidence interval; CVD: Cardiovascular disease; METS-VF: Metabolic score for visceral fat; LDL-C: Low-density lipoprotein cholesterol; Ua: Uric acid

Multivariable-adjusted restricted cubic spline (RCS) analyses revealed a positive linear (*P* for non-linear = 0.532) association between METS-VF and new-onset CVD in CHARLS, whereas a non-linear (*P* for non-linear < 0.001) association was observed in the UKB (Fig. 1). Therefore, we identified an inflection point of 7.285 for new-onset CVD, with the risk increasing by 30% per 1-SD increase in METS-VF up to this inflection point (Table S6). However, beyond this inflection point, each 1-SD increase in METS-VF was associated with a 1.45-fold increase in risk.

We further investigated the various CVD endpoint events as mentioned above using RCS analyses. In the UKB, RCS analyses revealed non-linear associations between METS-VF and IHD (*P* for non-linear < 0.001), angina (*P* for non-linear = 0.006), stroke (*P* for non-linear = 0.013), ISS (*P* for non-linear = 0.017), hemorrhagic stroke (*P* for non-linear = 0.029), AF (*P* for non-linear < 0.001), and HF (*P* for non-linear < 0.001); the inflection points were 7.244, 7.244, 7.213, 7.225, 6.928, 7.361, and 7.440, respectively. However, a positive linear association was observed between METS-VF and MI (Fig. S3, Table S6). In CHARLS, RCS analyses revealed a positive linear association between METS-VF and heart disease (*P* for non-linearity = 0.629), as well as a non-linear association between METS-VF and stroke (P for non-linearity = 0.005). The inflection point for stroke was 7.068 (Fig. S4, Table S7). In both UKB and CHARLS, the risk of new-onset CVD increased progressively with increasing METS-VF.

### Joint association of METS-VF and PRS with new-onset CVD

**Fig. 2:**
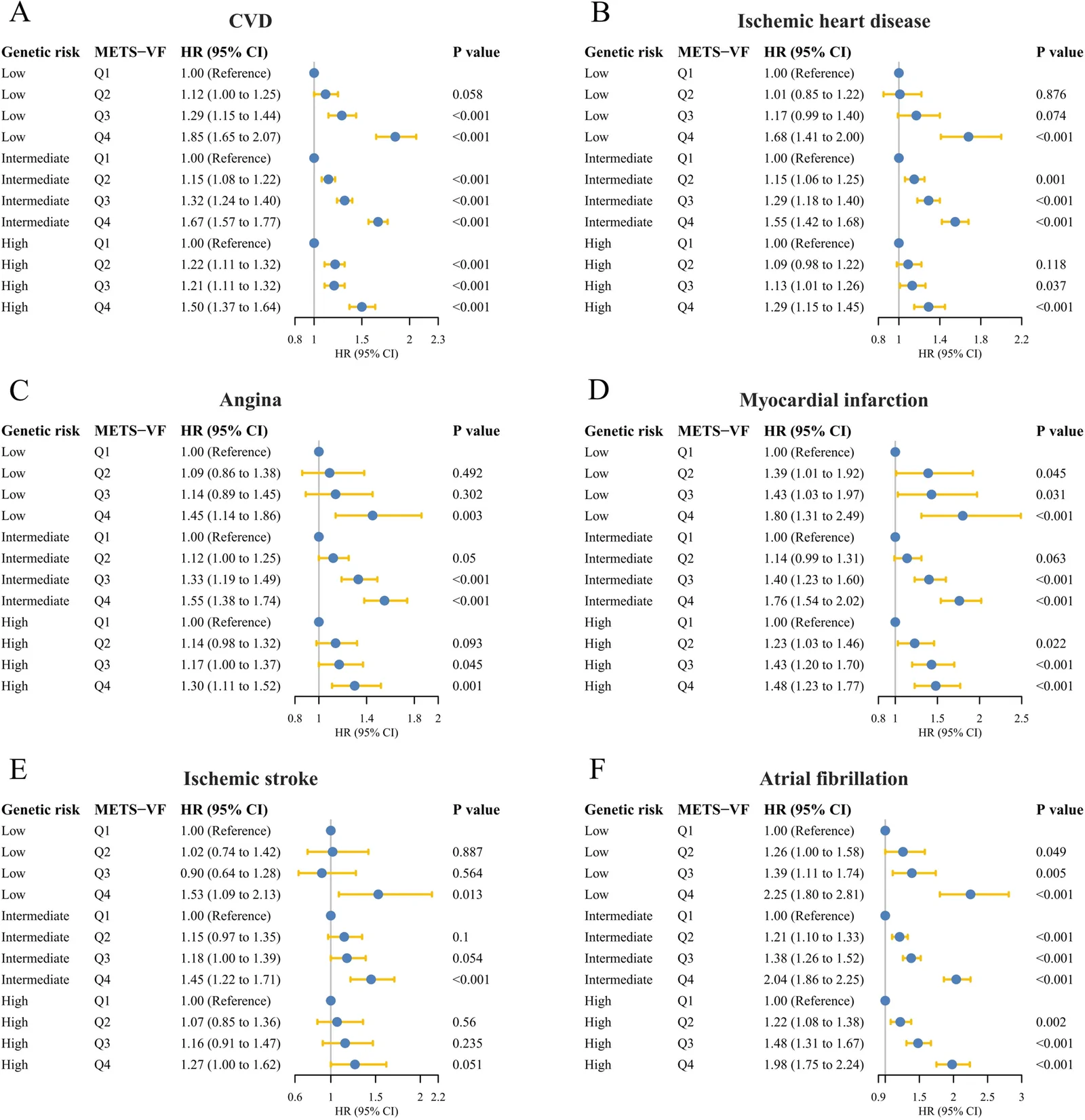
Association of METS-VF and PRS with CVD (**A**), IHD (**B**), angina (**C**), MI (**D**), ISS (**E**) and AF (**F**). Adjusted for age, gender, race, education level, smoking status, alcohol consumption status, physical activity, hypertension, diabetes, LDL-C, Ua and HbA1c. Abbreviations: HR: Hazard ratios; CI: Confidence interval; METS-VF: Metabolic score for visceral fat; CVD: Cardiovascular disease; IHD: Ischemic heart disease; MI: Myocardial infarction; ISS: Ischaemic stroke; AF: Atrial fibrillation; LDL-C: Low-density lipoprotein cholesterol; Ua: Uric acid

**Fig. 3:**
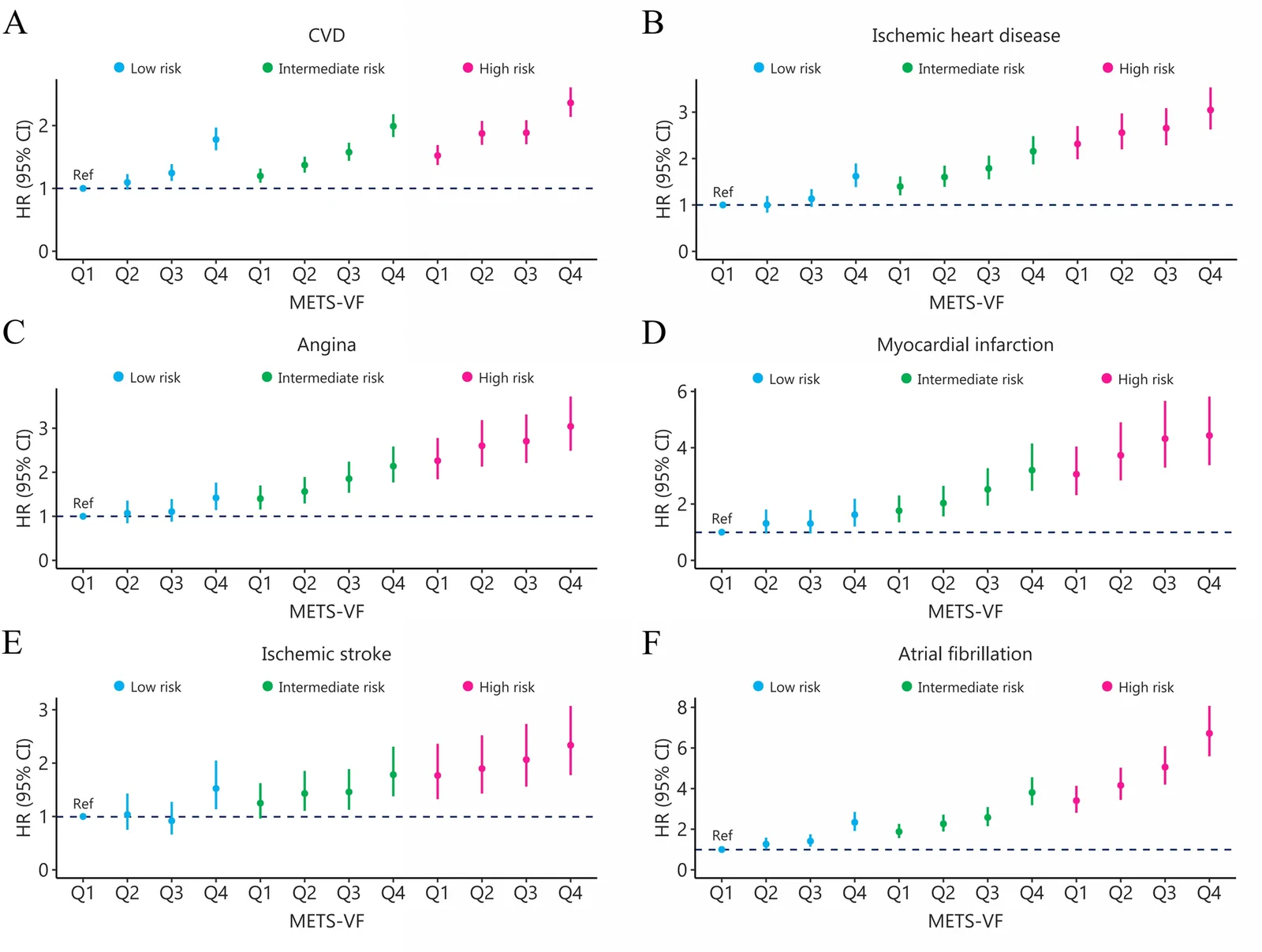
Joint Association of METS-VF and PRS with CVD (**A**), IHD (**B**), angina (**C**), MI (**D**), ISS (**E**) and AF (**F**). Adjusted for age, gender, race, education level, smoking status, alcohol consumption status, physical activity, hypertension, diabetes, LDL-C, Ua and HbA1c. Abbreviations: HR: Hazard ratios; CI: Confidence interval; METS-VF: Metabolic score for visceral fat; CVD: Cardiovascular disease; IHD: Ischemic heart disease; MI: Myocardial infarction; AF: Atrial fibrillation; LDL-C: Low-density lipoprotein cholesterol; Ua: Uric acid

Compared with low genetic risk, high genetic risk was associated with an increased risk of CVD (HR = 1.59, 95% CI: 1.52–1.66), IHD (HR = 2.32, 95% CI: 2.18–2.47), angina (HR = 2.39, 95% CI: 2.19–2.62), MI (HR = 3.18, 95% CI: 2.84–3.57), ISS (HR = 2.01, 95% CI: 1.77–2.28), and AF (HR = 3.36, 95% CI: 3.12–3.62), respectively (Table 2). Acquired environmental factors and genetic factors jointly contributed to the risk of CVD. Therefore, we further examined the association between METS-VF and new-onset CVD across different genetic risk groups. Figure 2 shows that among participants at high genetic risk, the highest quartile of METS-VF was associated with an increased risk of CVD (HR = 1.50, 95% CI: 1.37–1.64), IHD (HR = 1.29, 95% CI: 1.15–1.45), angina (HR = 1.30, 95% CI: 1.11–1.52), MI (HR = 1.48, 95% CI: 1.23–1.77), and AF (HR = 1.98, 95% CI: 1.75–2.24) in all high genetic risk groups, with the exception of IS (*P* = 0.051). Notably, Q2-Q4 quartiles of METS-VF were associated with the increased risk of incident AF in all genetic risk groups. In addition, individuals with high METS-VF and high genetic risk exhibited a significantly increased risk of new-onset CVD (HR = 2.36, 95% CI: 2.14–2.61), IHD (HR = 3.05, 95% CI: 2.63–3.53), angina (HR = 3.04, 95% CI: 2.49–3.72), MI (HR = 4.43, 95% CI: 3.38–5.82), ISS (HR = 2.34, 95% CI: 1.77–3.07), and AF (HR = 6.72, 95% CI: 5.59–8.72) compared with those with low METS-VF and low genetic risk (Fig. 3, Table S8).

**Table 2:** The associations between PRS and risk of new on-set CVD in UKB

**Variables**	**Case, n%**	** Model1**	**Model2**	**Model3**			
	***HR(95%CI)***	***P-value***	***HR(95%CI)***	***P-value***	***HR(95%CI)***	***P-value***	
CVD, *n*=20938 (20.96)							
PRS							
Low	3510/19975 (17.57)	Ref		Ref		Ref	
Intermediate	12462/59925 (20.80)	1.20 (1.16-1.25)	<0.001	1.25 (1.20-1.30)	<0.001	1.23 (1.19-1.28)	<0.001
High	4966/19979 (24.86)	1.48 (1.42-1.55)	<0.001	1.63 (1.56-1.70)	<0.001	1.59 (1.52-1.66)	<0.001
*P *for trend			<0.001		<0.001		<0.001
IHD, *n*=10869 (10.88)							
PRS							
Low	1481/19975 (7.41)	Ref		Ref		Ref	
Intermediate	6321/59925 (10.55)	1.45 (1.37-1.53)	<0.001	1.51 (1.43-1.60)	<0.001	1.49 (1.41-1.57)	<0.001
High	3067/19979 (15.35)	2.16 (2.03-2.30)	<0.001	2.37 (2.23-2.52)	<0.001	2.32 (2.18-2.47)	<0.001
*P *for trend			<0.001		<0.001		<0.001
Angina, *n*=5600 (5.60)							
PRS							
Low	719/19975 (3.60)	Ref		Ref		Ref	
Intermediate	3257/59925 (5.44)	1.53 (1.41-1.65)	<0.001	1.56 (1.44-1.70)	<0.001	1.55 (1.43-1.68)	0.010
High	1624/19979 (8.13)	2.31 (2.12-2.52)	<0.001	2.44 (2.24-2.67)	<0.001	2.39 (2.19-2.62)	<0.001
*P* for trend			<0.001		<0.001		<0.001
MI, *n*=3815 (3.81)							
PRS							
Low	401/19975 (2.01)	Ref		Ref		Ref	
Intermediate	2225/59925 (3.71)	1.87 (1.68-2.07)	<0.001	1.16 (1.06-1.28)	0.002	1.89 (1.70-2.10)	0.010
High	1189/19979 (5.95)	3.02 (2.70-3.39)	<0.001	1.34 (1.19-1.50)	<0.001	3.18 (2.84-3.57)	<0.001
*P* for trend			<0.001		<0.001		<0.001
ISS, *n*=2556 (2.56)							
PRS							
Low	360/19975 (1.80)	Ref		Ref		Ref	
Intermediate	1497/59925 (2.50)	1.39 (1.24-1.56)	<0.001	1.42 (1.27-1.60)	<0.001	1.39 (1.24-1.56)	0.010
High	699/19979 (3.50)	1.95 (1.72-2.21)	<0.001	2.09 (1.84-2.38)	<0.001	2.01 (1.77-2.28)	<0.001
*P* for trend			<0.001		<0.001		<0.001
AF, *n*=8861 (8.88)							
PRS							
Low	953/19975 (4.77)	Ref		Ref		Ref	
Intermediate	4983/59925 (8.32)	1.78 (1.66-1.90)	<0.001	1.80 (1.68-1.93)	<0.001	1.78 (1.66-1.91)	<0.001
High	2925/19979 (14.64)	3.24 (3.02-3.49)	<0.001	3.40 (3.16-3.66)	<0.001	3.36 (3.12-3.62)	<0.001
*P* for trend			<0.001		<0.001		<0.001

Model 1: Unadjusted. Model 2: Adjusted for age, gender, race, education, smoking, alcohol consumption, and physical activity. Model 3: included all variables from Model 2, and further adjusted for hypertension, diabetes, LDL-C, Ua and HbA1c

*Abbreviations*: *METS-VF *Metabolic score for visceral fat, *CVD* Cardiovascular disease, *HR* Hazard ratios, *CI* Confidence interval, *PRS* Polygenic risk score, *Ref* Reference, *IHD* Ischemic heart disease, *MI* Myocardial infarction, *ISS* Ischemic stroke, *AF* Atrial fibrillation, *LDL-C* Low-density lipoprotein cholesterol, *Ua* Uric acid

### Comparison of the predictive capability of various visceral fat indices and METS-VF for new-onset CVD

**Fig. 4:**
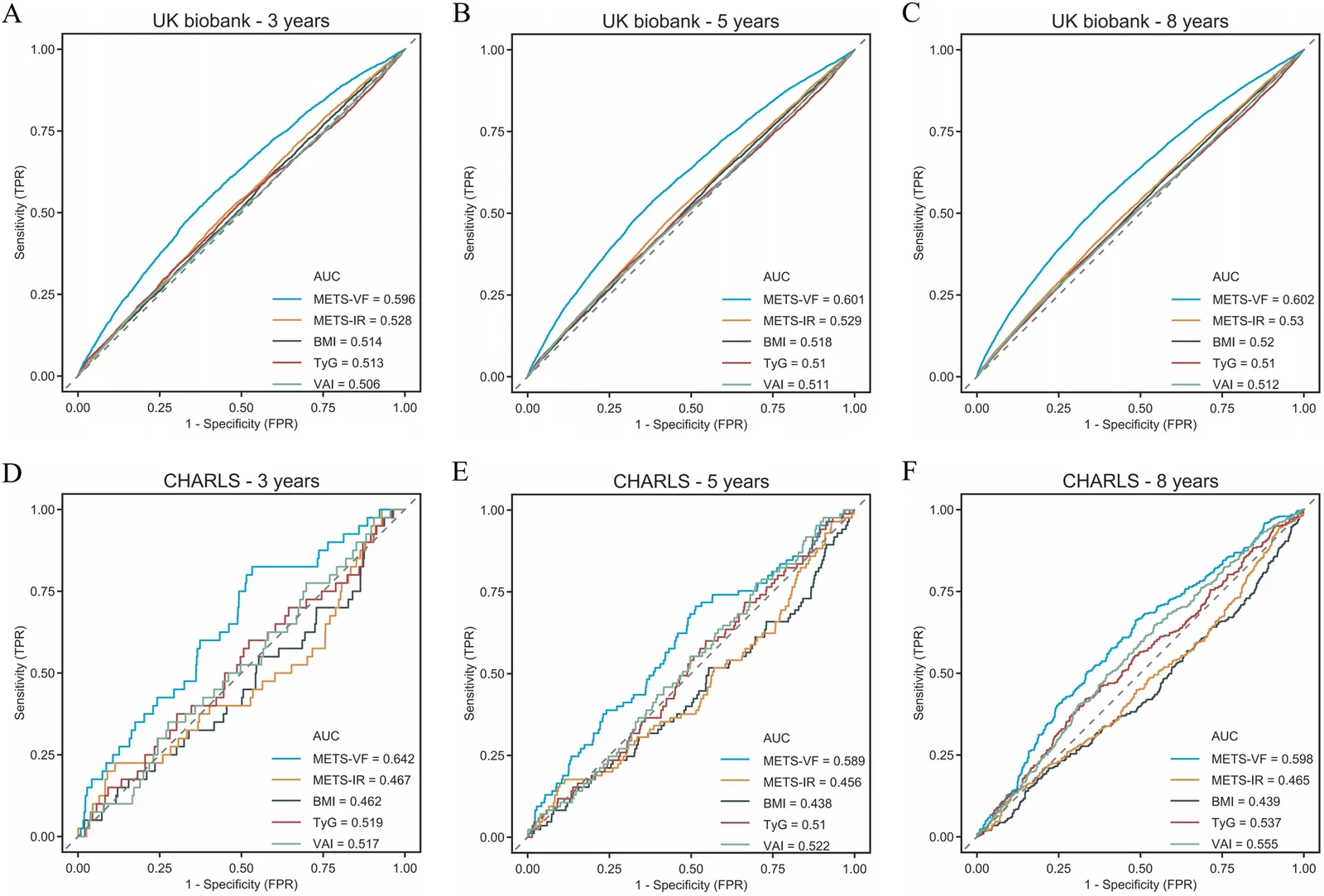
Receiver operating characteristics curve analysis for METS-VF to discriminate the 3-, 5-, and 8- years new-onset CVD. **A** UK Biobank, 3-year incident CVD; **B** UK Biobank, 5-year incident CVD; **C** UK Biobank, 8-year incident CVD; **D** CHARLS, 3-year incident CVD; **E** CHARLS, 5-year incident CVD; **F** CHARLS, 8-year incident CVD. AUC values for each index are shown in the inset of the corresponding panel. Abbreviations: AUC: Area under curve; CVD: Cardiovascular disease; METS-VF: Metabolic score for visceral fat; METS-IR: Metabolic score for insulin resistance; BMI: Body mass index (BMI); TyG: Triglyceride-glucose index; VAI: Visceral adiposity index

In participants with MetS, ROC curve analysis demonstrated that METS-VF exhibited the highest predictive capability for 3-year (AUC = 0.596 in UKB; AUC = 0.642 in CHARLS), 5-year (AUC = 0.601 in UKB; AUC = 0.589 in CHARLS), and 8-year (AUC = 0.602 in UKB; AUC = 0.598 in CHARLS) new-onset CVD, surpassing METS-IR, BMI, TyG, and VAI. The predictive effects of different visceral fat indices on new-onset CVD are illustrated in Fig. 4. Moreover, compared with TyG, VAI, BMI, and METS-IR, METS-VF significantly improved the NRI and IDI for new-onset CVD at 3-year, 5-year, and 8-year time points in both UKB and CHARLS (Table S9).

### Subgroup analysis

**Fig. 5:**
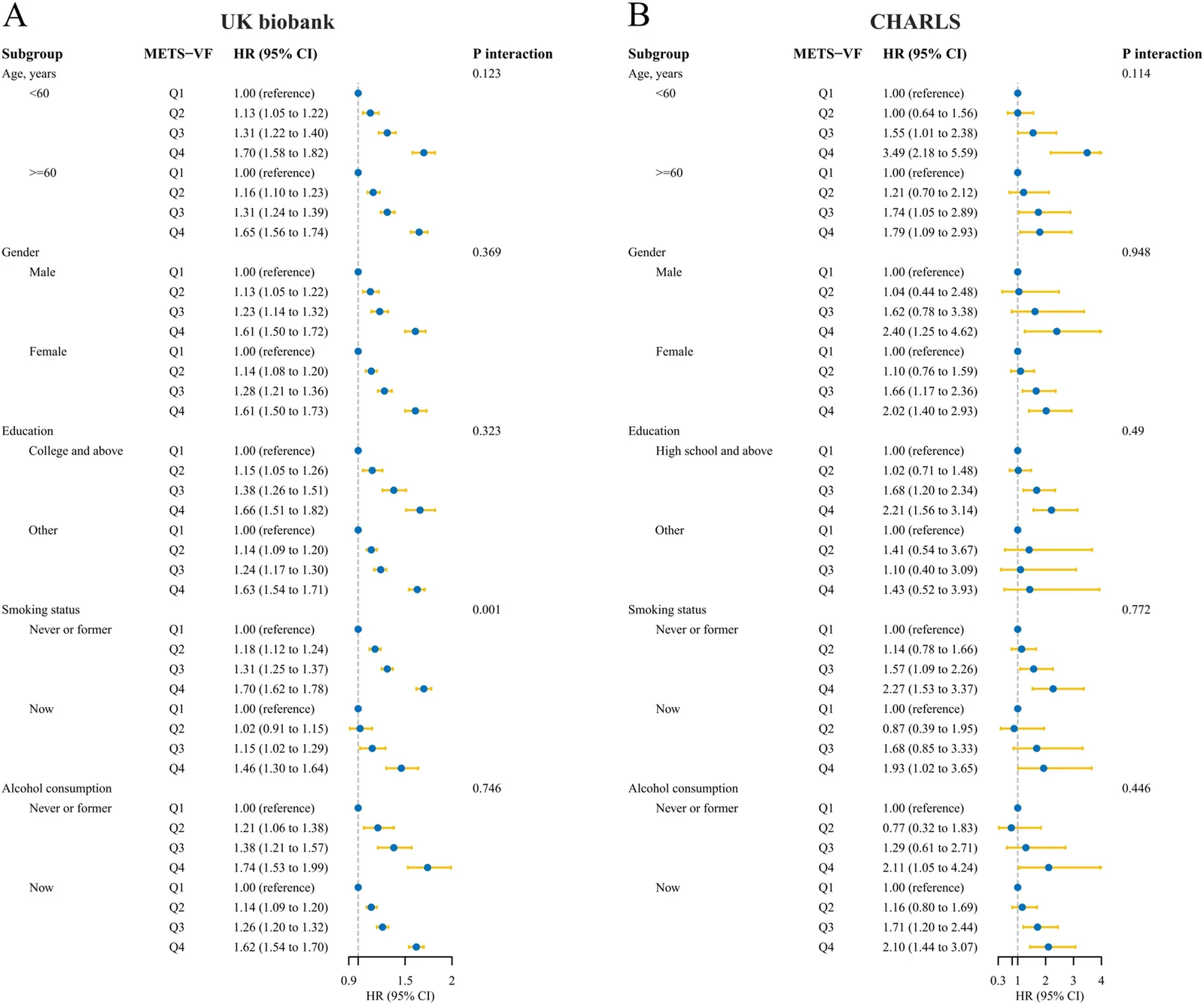
Subgroup and interaction analyses between METS-VF and new-onset CVD across age, gender, education, smoking status, and alcohol consumption subgroups. **A** UK Biobank cohort; **B** CHARLS cohort. HRs (95% CIs) were estimated using Cox proportional hazards models with the Q1 group as the reference. Adjusted for age, gender, race, education, smoking, alcohol consumption, moderated physical activity, hypertension, diabetes, LDL-C, Ua, and HbA1c. Abbreviations: HR: Hazard ratios; CI: Confidence interval; METS-VF: Metabolic score for visceral fat; CVD: Cardiovascular disease; AF: Atrial fibrillation; LDL-C: Low-density lipoprotein cholesterol; Ua: Uric acid

In the subgroup analysis, no significant interaction with new-onset CVD risk was observed in any subgroups in the UKB and CHARLS cohorts, except for smoking status (*P* = 0.001) in UKB (Fig. 5). Notably, among individuals who were never smokers or former smokers, those in the highest quartile of METS-VF exhibited a 70% increased risk compared with those in the lowest quartile (HR = 1.70, 95% CI: 1.62–1.78). However, this increase was only 46% in current smokers (HR = 1.46, 95% CI: 1.30–1.64).

### Competing risk analysis

In the UKB cohort, METS-VF exhibited a linear positive association with CVD risk when modeled as a continuous variable (per 1 SD increment: sHR = 1.58, 95% CI: 1.51–1.66). Categorical modeling by quartiles demonstrated a significant monotonic dose-response relationship, with the risk of CVD increasing progressively from Q1 to Q4 (*P* for trend < 0.001); participants in the highest quartile had a 56.8% higher risk of incident CVD relative to those in the lowest quartile (sHR = 1.57, 95% CI: 1.50–1.64) (Table S10).

In the CHARLS cohort, consistent with the UKB findings, METS-VF was positively associated with CVD risk in both continuous (per 1 SD increment: sHR = 1.34, 95% CI: 1.22–1.48) and categorical models. A significant dose-response gradient was observed across METS-VF quartiles (*P* for trend < 0.001), with the highest quartile associated with a more than 2-fold increase in CVD risk compared with the reference group (sHR = 2.04, 95% CI: 1.47–2.83) (Table S10).

### Sensitivity analysis

We conducted two sequential sensitivity analyses to verify the robustness of the primary competing risk results. First, we excluded metabolic variables. In the UKB cohort, each 1 SD increase in METS-VF was associated with higher CVD risk (HR = 1.71, 95% CI: 1.17–1.28), with a significant dose-response trend across METS-VF quartiles (*P* for trend < 0.001). In the CHARLS cohort, the corresponding HR was 1.52 (95% CI: 1.38–1.69). Elevated CVD risk was observed in Q3 and Q4, while Q2 showed no significant difference versus the reference group (*P* = 0.345) (Table S11).

We further excluded individuals taking medications, leaving 47,006 participants in the UKB cohort and 930 in the CHARLS cohort. The association between METS-VF and incident CVD remained statistically significant in this restricted cohort: the HR was 1.78 (95% CI: 1.65–1.92) for UKB and 1.35 (95% CI: 1.16–1.57) for CHARLS. The dose-response trend across quartiles remained significant in both cohorts (Table S12).

Overall, these findings verify that the observed association is not affected by baseline cardiometabolic disorders and related medications, further proving the robustness of our results.

## Discussion

In this study involving two prospective cohorts, we identified an independent association between METS-VF and an increased risk of new-onset CVD in patients with MetS, even after adjustment for traditional cardiovascular risk factors. Higher METS-VF was associated with a higher risk of new-onset CVD. Moreover, individuals with high METS-VF and high genetic risk exhibited a significantly increased risk of new-onset CVD. Competing risk analysis and sensitivity analyses further confirmed the robustness of the association. These finding support METS-VF as a valuable biomarker for cardiovascular risk stratification in MetS populations.

Previous studies have demonstrated that visceral fat plays a critical role in the development of MetS and CVD [14, 18, 34]. Excessive visceral fat accumulation triggers the release of numerous adipokines and inflammatory mediators [35, 36], which contribute to insulin resistance, dyslipidemia, and endothelial dysfunction, ultimately elevating the risk of CVD [37–39]. A previous study suggested that METS-VF is an independent risk factor for cardiovascular mortality (HR = 2.62, 95% CI: 1.20–5.71), and for every 0.2-unit increase in METS-VF, cardiovascular mortality increases by 18% (HR = 1.18, 95% CI: 1.06–1.31) [40]. Furthermore, a study from CHARLS demonstrated a dose-response relationship between METS-VF and CVD events (HR = 3.31, 95% CI: 1.28–8.54) as well as all-cause mortality (HR = 4.03, 95% CI: 1.72–9.42) across different glucose tolerance statuses [41]. METS-VF, as a comprehensive indicator reflecting the metabolic status of visceral fat, integrates multiple parameters associated with visceral fat metabolism [42]. A cohort study in China involving 41,576 individuals found that high cumulative METS-VF was associated with an increased risk of CVD (HR = 2.78, 95% CI: 2.49–3.17) and mortality (HR = 4.90, 95% CI: 4.36–5.50), with this association becoming stronger as exposure to high METS-VF was prolonged [43]. These studies suggest that a higher METS-VF signifies a more pronounced metabolic disturbance in visceral fat, likely explaining its association with an elevated risk of new-onset CVD. Our findings are consistent with previous studies highlighting the importance of visceral fat metabolism in the pathogenesis of CVD [14, 18, 25]. Additionally, this study is distinct in its use of METS-VF combined with PRS to comprehensively evaluate the relationship between visceral fat metabolism and new-onset CVD in patients with MetS. The results indicate that METS-VF is associated with the risk of new-onset cardiovascular disease in patients with MetS, while genetic risk also plays a vital role.

Consistent with a prior report by Duan et al., which documented an independent association between elevated METS-VF and incident AF (adjusted HR = 1.38, 95% CI 1.34–1.42) [44], we further demonstrated that participants with both high METS-VF and high genetic risk exhibited a 6.72-fold greater AF risk relative to those with low METS-VF and low genetic susceptibility. This indicates that adverse metabolic and genetic profiles augment CVD risk, whereas healthy lifestyles and favorable metabolic status alleviate inherited disease susceptibility across all ages [45]. Since MetS itself confers robust AF risk and optimized MetS management lowers AF burden and improves rhythm-control outcomes [46], targeted visceral fat monitoring via METS-VF may help reduce incident AF among individuals with MetS, though additional prospective investigations are needed to confirm this therapeutic implication.

RCS analysis identified a non-linear dose-response in UKB, with an inflection point at 7.285. Below this value, each 1-SD increase in METS-VF was associated with a 30% higher CVD risk; beyond it, the same increment carried a 1.45-fold risk. This accelerating curve implies a metabolic tipping point: once METS-VF exceeds 7.285, adipose tissue-derived inflammatory mediators, free fatty acids, and adipokine imbalance may initiate a self-amplifying cascade of insulin resistance and endothelial dysfunction, driving rapid cardiovascular deterioration [15, 25, 38, 43]. The linear association observed in CHARLS likely reflects limited power from the smaller sample (*n* = 1,680) and shorter follow-up (5.0 years), which may mask long-term non-linear trajectories. Clinically, a METS-VF cut-off of 7.285 may help stratify risk in MetS. Patients with values between 6.0 and 7.0 could benefit most from early intervention, as measures targeting visceral fat metabolism may have greater preventive impact before this boundary is crossed [6, 42].

In addition, there was an interaction between smoking and METS-VF on new-onset CVD risk in the UKB cohort. It is plausible that MetS and smoking contribute to CVD risk through shared pathophysiological pathways, such as reduced insulin sensitivity, impaired glycemic control, altered lipid profiles, and endothelial dysfunction [47, 48]. A previous study indicated that genetic predisposition toward tobacco smoking was strongly associated with a higher likelihood of MetS (HR = 1.49, 95% CI: 1.47–1.52) in Chinese individuals [49]. However, our study revealed that the risk of new-onset CVD increased in both never and former smokers as well as in current smokers as METS-VF rose in the UKB cohort. The magnitude of this increased risk was more pronounced among never smokers or former smokers. In current smokers, multiple other strong cardiovascular risk factors may mask the marginal effect of METS-VF [50]. Meanwhile, we acknowledge that residual confounding may partially explain this counterintuitive interaction finding.

Both METS-IR, BMI, TyG, and VAI have been used to evaluate the risk of CVD in patients with MetS [23, 51, 52]. Recently, Wang et al. [53] found that METS-VF was strongly associated with left ventricular hypertrophy in T2DM (OR = 9.79, 95% CI: 6.16–15.76), demonstrating more favorable predictive performance compared to traditional indices (AUC = 0.68, 95% CI: 0.66–0.70). Cao et al. [54] found that METS-VF was independently associated with the risk of stroke (HR = 2.78, 95% CI: 1.71–4.52) and exhibited the highest AUC for stroke prediction (AUC = 0.687, 95% CI: 0.668–0.706), outperforming BMI, WHtR, VAI, and cardiometabolic index (CMI). METS-VF offered predictive value beyond traditional indices, with NRI (0.125–0.257) and IDI (0.004–0.022) consistently positive and statistically significant across 3-, 5-, and 8-year follow-ups in both cohorts [55]. These improvements reflect clinically actionable risk stratification for patients with MetS, rather than isolated gains in statistical performance.

Even when conventional metabolic indicators are within normal ranges, patients with MetS remain at substantial cardiovascular risk. METS-VF integrates Glu, TG, HDL-C, age, gender and anthropometric measurements, yet it cannot account for residual cardiovascular risk factors including Lp (a) [56]. Emerging data indicate inflammation brings extra residual cardiovascular risks beyond routine metabolic markers. In familial hypercholesterolemia, LDLR mutation carriers present higher NLR and more severe subclinical atherosclerosis. Continuous LDL-C exposure activates innate immunity, a process undetectable by conventional metabolic indicators [57]. These limitation emphasize the importance of comprehensive risk evaluation, and METS-VF can serve as an essential component of the integrated risk stratification, further strengthening the translational value of our work. Since METS-VF is closely linked to visceral fat burden, individuals with elevated METS-VF should receive additional imaging assessment to quantify visceral fat volume [22, 42]. Relevant interventions are expected to mitigate long-term cardiometabolic risks in this population [58, 59]. Specifically, combined intensive lifestyle adjustments (e.g., dietary management and regular physical activity) and targeted pharmacotherapies are recommended to improve visceral fat metabolism and reduce the risk of new-onset CVD [60, 61].

The present study originates from two rigorously designed prospective cohorts of different ethnic backgrounds with prolonged follow-up, and consistent results across the two cohorts strengthen the external validity of our observations. Nevertheless, this study has certain limitations. First, while the sample size of UK Biobank is sufficient, the sample size of CHARLS is relatively limited and may not encompass all clinical scenarios and population characteristics, which might have introduced some bias into the study results. Competing risks analysis and sensitivity analyses confirms stable primary effect estimates; however, the findings for subgroup analysis should be interpreted with caution. Second, outcome assessment approaches differ substantially between cohorts. CHARLS relies on self-reported physician-confirmed CVD diagnoses, which are vulnerable to recall bias, differential misclassification and underascertainment, restricting granular endpoint analyses and cross-cohort comparisons. Third, dynamic blood sampling was not performed during the UK Biobank follow-up period, and in CHARLS, blood samples were collected only at baseline, wave 3, and wave 5. This limitation restricted our ability to further investigate the relationship between changes in METS-VF and new-onset CVD. Fourth, although we adjusted for traditional cardiovascular risk factors, unmeasured confounding factors (e.g., unmeasured lifestyle variables) might still influence the association between METS-VF and new-onset CVD. Fifth, measurement error associated with the calculation of METS-VF-related indicators. Finally, in addition to the European and Asian populations, it may also be necessary to confirm the universality across other ethnic subgroups.

## Conclusions

The present study indicated that an elevated METS-VF was significantly associated with new-onset CVD among patients with MetS. Individuals with high METS-VF were at increased risk of new-onset CVD, whereas those with both high genetic risk and high METS-VF demonstrated the highest risk. METS-VF may serve as a more effective and independent predictor of CVD in this population.

## Supplementary Information

Supplementary Material 1.

## Data Availability

The data are available on application through approval and oversight by the UK Biobank (www.ukbiobank.ac.uk/). The datasets used in this investigation are available in online repositories (http://charls.pku.edu.cn//).

## Abbreviations

MetS: metabolic syndrome
CVD: cardiovascular disease
METS-VF: metabolic score for visceral fat
UKB: UK Biobank
CHARLS: China Health and Retirement Longitudinal Study
RCS: restricted cubic spline
PRS: Polygenic risk score
ROC: receiver operating characteristic
NRI: net reclassification improvement
IDI: integrated discrimination improvement
TNF-α: tumour necrosis factor-alpha
IL-6: interleukin-6
METS-IR: metabolic score for insulin resistance
WHtR: waist-to-height ratio
BMI: body mass index
WC: waist circumference
TyG: triglyceride-Glucose index
VAI: visceral adiposity index
MREC: Multi-centre Research Ethics Committee
RTB: Research Tissue Bank
MI: myocardial infarction
ISS: ischemic stroke
AF: atrial fibrillation
HF: heart failure
FPG: fasting plasma glucose
IHD: ischemic heart disease
CAD: coronary artery disease
GWAS: genome-wide association study
SD: standard deviation
