# Supplementary material for: Metabolic score for visceral fat as a predictor of new-onset cardiovascular disease in patients with metabolic syndrome: a validation study in two prospective cohorts (UK Biobank and CHARLS)
Source: BMC Cardiovasc Disord. 2026 Aug 1;26:992. doi: 10.1186/s12872-026-06299-2 (PMC13718111; doi:10.1186/s12872-026-06299-2)
Supplement: Supplementary file 1 [file 12872_2026_6299_MOESM1_ESM.pdf]

Table S1 Missing data patterns for covariates in the UK Biobank and CHALRS cohort

Variable	Missing rate (%)
**UK Biobank**	
HbA1c	7.2
LDL-C	6.7
Ua	6.7
Smoking status (%)	0.6
Education level(%)	0.6
Alcohol consumption status(%)	0.3
Physical activity frequency	0.2
Age	0
Gender (%)	0
Race-white (%)	0
Hypertension (%)	0
Diabetes (%)	0
**CHALRS**	
Smoking status (%)	2.3
HbA1c	0.9
Alcohol consumption status(%)	0.5
LDL-C	0.2
Age	0
Ua	0
Age	0
Education level(%)	0
Hypertension (%)	0
Diabetes (%)	0

Missing values were addressed using Multiple Imputation by Chained Equations (MICE), generating 5 imputed datasets. Abbreviations: LDL-C: Low-density lipoprotein cholesterol; Ua: Uric acid.

Table S2 Demographic characteristics of the study participants in UK Biobank

Characteristic	Total	METS-VF				*P value*
		Q1 (<6.916)	Q2 (6.916-7.207)	Q3 (7.207-7.449)	Q4 (>7.449)	
Participants	n=101292	n=25323	n=25323	n=25323	n=25323	
Age, years	57.826±7.684	56.615±7.856	57.346±7.856	57.801±7.593	59.543±6.916	< 0.001
Gender, male, n (%)	50469 (49.83)	5356 (21.15)	9482 (37.44)	15297 (60.41)	20334 (80.30)	< 0.001
BMI, kg/m2	31.069±4.855	26.956±2.888	29.967±3.052	31.931±3.673	35.421±5.033	< 0.001
Height, cm	168.708±9.578	166.34±8.443	167.294±10.196	169.986±10.05	171.214±8.673	< 0.001
WC, cm	100.799±11.77	87.673±6.309	96.993±5.068	104.199±5.201	114.33±8.955	< 0.001
SBP, mmHg	149.100±18.224	147.677±18.700	148.237±18.206	149.540±17.818	150.963±17.983	< 0.001
DBP, mmHg	89.011±9.769	87.836±9.635	88.693±9.634	89.689±9.626	89.834±10.041	< 0.001
HbA1c, (%)	5.537 (5.281-5.857)	5.464 (5.226-5.729)	5.509 (5.271-5.802)	5.537 (5.281-5.857)	5.656 (5.363-6.141)	< 0.001
Hypertention, n (%)	70417 (69.52)	16282 (64.30)	16784 (66.28)	17996 (71.07)	19355 (76.43)	< 0.001
Education level, n (%)						< 0.001
College or above	26615 (26.28)	7489 (29.57)	6781 (26.78)	6547 (25.85)	5798 (22.90)	
others	74677 (73.72)	17834 (70.43)	18542 (73.22)	18776 (74.15)	19525 (77.10)	
Race						< 0.001
White	95017 (93.81)	23563 (93.05)	23552 (93.01)	23715 (93.65)	24187 (95.51)	
Latin	524 (0.52)	150 (0.59)	124 (0.49)	133 (0.53)	117 (0.46)	
Black	4077 (4.02)	1213 (4.79)	1153 (4.55)	1015 (4.01)	696 (2.75)	
Asian	1674 (1.65)	397 (1.57)	494 (1.95)	460 (1.82)	323 (1.28)	
Smoking status, n (%))						< 0.001
Never	51616 (50.96)	14462 (57.11)	13749 (54.29)	12517 (49.43)	10888 (43.00)	
Former	38374 (37.88)	7907 (31.22)	8820 (34.83)	9974 (39.39)	11673 (46.10)	
Now	11302 (11.16)	2954 (11.67)	2754 (10.88)	2832 (11.18)	2762 (10.91)	
Alcohol consumption status, n (%)						< 0.001
Never	5708 (5.64)	1520 (6.00)	1629 (6.43)	1410 (5.57)	1149 (4.54)	
Former	4339 (4.28)	965 (3.81)	1031 (4.07)	1065 (4.21)	1278 (5.05)	
Now	91245 (90.08)	22838 (90.19)	22663 (89.50)	22848 (90.23)	22896 (90.42)	
Physical activity frequency , n (%)						< 0.001
Never	16515 (16.30)	3333 (13.16)	3884 (15.34)	4275 (16.88)	5023 (19.84)	
< 3 times per week	38934 (38.44)	9796 (38.68)	9885 (39.04)	9775 (38.60)	9478 (37.43)	
≥ 3 times per week	45843 (45.26)	12194 (48.15)	11554 (45.63)	11273 (44.52)	10822 (42.74)	
Lipid-lowing medication, n (%)	34413 (33.97)	7745 (30.58)	8199 (32.38)	8541 (33.73)	9928 (39.21)	< 0.001
Blood pressure medication, n (%)	39439 (38.94)	8280 (32.70)	9027 (35.65)	9921 (39.18)	12211 (48.22)	< 0.001

Aspirin, n (%)	18017 (17.79)	3424 (13.52)	3873 (15.29)	4542 (17.94)	6178 (24.40)	< 0.001
Insulin, n (%)	2398 (2.37)	525 (2.07)	453 (1.79)	501 (1.98)	919 (3.63)	< 0.001
Anticoagulant	130 (0.13)	28 (0.11)	20 (0.08)	32 (0.13)	50 (0.20)	0.002
Laboratory assessments						
Scr, umol/L	71.6 (62.1-82.3)	65.9 (58.5-75.2)	69.3 (60.5-79.9)	74.3 (64.3-84.4)	77.2 (67.9-87.1)	< 0.001
eGFR, ml/min	86.964 (77.129-96.338)	90.003 (80.235-99.334)	87.588 (77.735-96.960)	86.514 (76.784-95.580)	83.727 (74.192-92.628)	< 0.001
Ua, umol/L	337.5 (285.8-393.3)	296.2 (252.8-344.0)	327.2 (280.3-378.1)	354.1 (306.3-405.3)	374.2 (323.6-426.8)	< 0.001
Cys-C	0.935 (0.849-1.035)	0.882 (0.803-0.973)	0.919 (0.840-1.013)	0.947 (0.867-1.043)	0.993 (0.905-1.099)	< 0.001
TC, mmol/L	5.569 (4.763-6.437)	5.748 (4.976-6.608)	5.697 (4.892-6.57)	5.564 (4.773-6.421)	5.246 (4.432-6.102)	< 0.001
TG, mg/dL	196.736 (156.645-261.872)	186.292 (154.167-240.499)	193.284 (155.672-255.234)	201.869 (158.327-270.368)	209.037 (160.274-283.51)	< 0.001
LDL-C, mmol/L	3.532 (2.893-4.194)	3.645 (3.008-4.300)	3.623 (2.983-4.287)	3.535 (2.912-4.196)	3.326 (2.692-3.976)	< 0.001
HDL-C, mg/dL	46.056 (39.250-53.519)	48.956 (43.117-58.198)	47.525 (40.758-55.491)	45.399 (38.902-52.398)	41.996 (36.504-48.415)	< 0.001
GLU, mg/dL	93.150 (85.338-105.084)	93.438 (85.446-105.030)	92.394 (85.050-103.662)	92.430 (84.978-103.77)	94.446 (86.004-109.386)	< 0.001
METS-VF	7.158±0.420	6.602±0.319	7.069±0.083	7.328±0.069	7.634±0.145	< 0.001

The chi-square test was used for the comparison of categorical variables; the *t*-test was employed to compare variates with normal distribution, and the rank-sum test was conducted for variates with skewed distribution. Abbreviations: METS-VF: Metabolic score for visceral fat; BMI: Body mass index; WC: Waist circumference; SBP: Systolic blood pressure; DBP: Diastolic blood pressure; Ua: Uric acid; BUN: Blood urea nitrogen; TC: Total cholesterol; TG: Triglycerides; HDL-C: High-density lipoprotein cholesterol; LDL-C: Low-density lipoprotein cholesterol; GLU: Glucose; Scr: Serum creatinine; eGFR: Estimated glomerular filtration rate; Cys-C:Cystatin C

Table S3 Demographic characteristics of the study participants in CHARLS

Characteristic	Total	METS-VF				***P value***
		**Q1 (<6.800)**	**Q2 (6.800-7.011)**	**Q3 (7.011-7.192)**	**Q4 (>7.192)**	
Participants	n=1680	n=420	n=420	n=420	n=420	

Age, years	59 (52-65)	55 (49-61)	58 (52-64)	60 (54-66)	63 (57-70)	< 0.001
Gender, male, n (%)	445 (26.49)	80 (19.05)	68 (16.19)	99 (23.57)	198 (47.14)	< 0.001
BMI, kg/m2	26.212 (23.825-28.463)	23.368 (21.476-25.333)	25.506 (23.606-27.439)	26.908 (25.116-28.644)	28.846 (26.829-30.863)	< 0.001
Height, cm	156.358±9.141	157.161±10.489	155.488±7.168	155.452±8.280	157.331±10.084	0.001
WC, cm	92.830±10.533	82.514±10.533	91.389±4.496	95.742±4.698	101.676±9.780	< 0.001
SBP, mmHg	140.667 (125.667-153.333)	138 (122.167-151.5)	138.667 (124.667-151)	141.333 (128.667-154.083)	142.167 (129.417-158.75)	< 0.001
DBP, mmHg	80.667 (72.33-89)	80.167 (71.67-90)	80.333 (73-88)	80.833 (73-89.25)	80.667 (72.33-89.33)	0.902
HbA1c, (%)	5.3 (5-5.7)	5.2 (4.9-5.6)	5.2 (4.9-5.6)	5.3 (5-5.8)	5.4 (5.1-5.9)	0.077
Hypertention, n (%)	1243 (73.99)	292 (69.52)	286 (68.10)	314 (74.76)	351 (83.57)	< 0.001
Dyslipidemia (%)	284 (17.38)	63 (15.52)	55 (13.38)	60 (14.71)	106 (25.92)	< 0.001
Diabetes (%)	727 (43.27)	198 (47.14)	165 (39.29)	167 (39.76)	197 (46.90)	0.022
Education level, n (%)						< 0.001
Senior high school or above	1470 (87.50)	366 (87.14)	359 (85.48)	370 (88.10)	375 (89.29)	0.394
others	210 (12.50)	54 (12.86)	61 (14.52)	50 (11.90)	45 (10.71)	
Smoking status, n (%))						< 0.001
Never or former	1269 (75.63)	340 (81.15)	344 (81.90)	317 (75.48)	268 (63.96)	
Now	409 (24.37)	79 (18.85)	76 (18.10)	103 (24.52)	151 (36.04)	
Alcohol consumption status, n (%)						0.005
Never or former	355 (21.13)	86 (20.48)	75 (17.86)	80 (19.05)	114 (27.14)	
Now	1325 (78.87)	334 (79.52)	345 (82.14)	340 (80.95)	306 (72.86)	
Lipid-lowing medication, n (%)	170 (10.12)	36 (8.57)	34 (8.10)	33 (7.86)	67 (15.95)	< 0.001
Blood pressure medication, n (%)	644 (38.33)	117 (27.86)	147 (35.00)	160 (38.10)	220 (52.38)	< 0.001
Medicine for diabetes (%)	160 (9.52)	27 (6.43)	40 (9.52)	39 (9.29)	54 (12.86)	0.018
Laboratory assessments						
Scr, umol/L	0.735 (0.644-0.859)	0.712 (0.61-0.802)	0.723 (0.644-0.831)	0.74 (0.641-0.870)	0.78 (0.655-0.927)	<0.001
eGFR, ml/min	84.913 (71.577-99.073)	94.294 (81.154-104.346)	86.06 (73.207-98.782)	84.596 (71.027-96.455)	74.905 (63.410-89.472)	< 0.001

Ua, umol/L	4.501 (3.708-5.384)	4.14 (3.499-5.032)	4.423 (3.641-5.192)	4.655 (3.720-5.316)	4.927 (4.119-5.952)	< 0.001
Cys-C, mmol/L	0.95 (0.810-1.098)	0.86 (0.750-0.990)	0.92 (0.810-1.050)	0.96 (0.830-1.100)	1.03 (0.903-1.190)	< 0.001
TC, mg/dL	199.486 (173.970-228.094)	196.200 (174.260-225.774)	198.326 (171.264-226.548)	203.738 (177.063-230.51)	199.486 (172.617-225.388)	0.615
TG, mg/dL	188.505 (146.025-259.526)	196.912 (160.185-260.411)	183.195 (136.069-248.685)	184.080 (136.29-258.862)	189.390 (141.821-265.942)	0.089
LDL-C, mg/dL	39.047 (33.248-45.619)	39.820 (34.021-44.846)	39.820 (34.407-46.779)	39.047 (33.248-46.102)	37.114 (30.928-43.686)	< 0.001
HDL-C, mg/dL	114.434 (90.464-141.012)	111.341 (89.305-137.243)	115.980 (89.498-143.042)	117.526 (92.784-143.429)	114.047 (86.598-138.016)	0.056
GLU, mg/dL	113.040 (101.160-139.14)	114.480 (100.980-139.59)	110.070 (100.080-135.945)	111.960 (100.62-138.96)	117.090 (103.32-144.18)	0.555
METS-VF	6.966±0.382	6.478±0.363	6.919±0.060	7.102±0.054	7.364±0.170	< 0.001

The chi-square test was used for the comparison of categorical variables; the t-test was employed to compare variates with normal distribution, and the rank-sum test was conducted for variates with skewed distribution. Abbreviations: METS-VF: Metabolic score for visceral fat; BMI: Body mass index; WC: Waist circumference; SBP: Systolic blood pressure; DBP: Diastolic blood pressure; Ua: Uric acid; TC: Total cholesterol; TG: Triglycerides; HDL-C: High-density lipoprotein cholesterol; LDL-C:Low-density lipoprotein cholesterol; GLU: Glucose; Scr: Serum creatinine; eGFR: Estimated glomerular filtration rate; Cys-C:Cystatin C.

Table S4 Associations of METS-VF with IHD, Angina, MI, stroke, ISS, hemorrhagic stroke, AF and HF in UK Biobank

Variable	Characteristics	Cases/Total(%)	*HR (95%CI)*	*P value*	*P for trend*
**IHD**					
Model1	Per 1 SD	11019/101292 (10.88)	2.28 (2.17–2.40)	< 0.001	
	Q1	1859/25323 (7.34)	Ref		< 0.001
	Q2	2312/25323 (9.13)	1.26 (1.19–1.34)	< 0.001	
	Q3	2868/25323 (11.33)	1.59 (1.50–1.69)	< 0.001	
	Q4	3980/25323 (15.72)	2.31 (1.19–2.44)	< 0.001	
Model2	Per 1 SD	11019/101292 (10.88)	1.66 (1.57–1.76)		
	Q1	1859/25323 (7.34)	Ref		< 0.001
	Q2	2312/25323 (9.13)	1.14 (1.07–1.22)	< 0.001	
	Q3	2868/25323 (11.33)	1.27 (1.20–1.36)	< 0.001	
	Q4	3980/25323 (15.72)	1.67 (1.57–1.77)	< 0.001	
Model3	Per 1 SD	11019/101292 (10.88)	1.44 (1.36–1.52)	< 0.001	
	Q1	1859/25323 (7.34)	Ref		< 0.001
	Q2	2312/25323 (9.13)	1.10 (1.04–1.17)	0.002	
	Q3	2868/25323 (11.33)	1.19 (1.12–1.27)	< 0.001	
	Q4	3980/25323 (15.72)	1.45 (1.36–1.55)	< 0.001	
**Angina**					
Model1	Per 1 SD		1.85 (1.73–1.98)	< 0.001	
	Q1	5673/101292 (5.60)	Ref		< 0.001
	Q2	1038/25323 (4.10)	1.21 (1.11–1.31)	< 0.001	
	Q3	1243/25323 (4.91)	1.46 (1.36–1.59)	< 0.001	
	Q4	1494/25323 (5.90)	1.92 (1.78–2.07)	< 0.001	
Model2	Per 1 SD	1898/25323 (7.50)	1.53 (1.42–1.65)	< 0.001	
	Q1	5673/101292 (5.60)	Ref		< 0.001
	Q2	1038/25323 (4.10)	1.14 (1.05–1.24)	0.002	
	Q3	1243/25323 (4.91)	1.29 (1.19–1.41)	< 0.001	
	Q4	1494/25323 (5.90)	1.59 (1.45–1.72)	< 0.001	
Model3	Per 1 SD	1898/25323 (7.50)	1.38 (1.28–1.49)	< 0.001	
	Q1	5673/101292 (5.60)	Ref		< 0.001
	Q2	1038/25323 (4.10)	1.11 (1.02–1.21)	0.012	
	Q3	1243/25323 (4.91)	1.23 (1.13–1.34)	< 0.001	
	Q4	1494/25323 (5.90)	1.43 (1.32–1.56)	< 0.001	

**MI**					
Model1	Per 1 SD	3860/101292 (3.81)	2.03 (1.87–2.21)	< 0.001	
	Q1	668/25323 (2.64)	Ref		< 0.001
	Q2	839/25323 (3.31)	1.27 (1.14–1.40)	< 0.001	
	Q3	1041/25323 (4.11)	1.59 (1.44–1.75)	< 0.001	
	Q4	1312/25323 (5.18)	2.06 (1.88–2.26)	< 0.001	
Model2	Per 1 SD	3860/101292 (3.81)	1.35 (1.23–1.48)	< 0.001	
	Q1	668/25323 (2.64)	Ref		
	Q2	839/25323 (3.31)	1.11 (1.00–1.23)	0.049	< 0.001
	Q3	1041/25323 (4.11)	1.18 (1.07–1.31)	0.001	
	Q4	1312/25323 (5.18)	1.34 (1.21–1.49)	< 0.001	
Model3	Per 1 SD	3860/101292 (3.81)	1.20 (1.09–1.32)	< 0.001	
	Q1	668/25323 (2.64)	Ref		< 0.001
	Q2	839/25323 (3.31)	1.07 (0.96–1.18)	0.218	
	Q3	1041/25323 (4.11)	1.11 (1.00–1.23)	0.056	
	Q4	1312/25323 (5.18)	1.20 (1.08–1.33)	0.001	
**Stroke**					
Model1	Per 1 SD	3093/101292 (3.05)	1.84 (1.67–2.01)	< 0.001	
	Q1	589/25323 (2.33)	Ref		< 0.001
	Q2	716/25323 (2.83)	1.22 (1.10–1.37)	< 0.001	
	Q3	751/25323 (2.97)	1.30 (1.16–1.44)	< 0.001	
	Q4	1037/25323 (4.10)	1.85 (1.67–2.04)	< 0.001	
Model2	Per 1 SD	3093/101292 (3.05)	1.66 (1.50–1.84)	< 0.001	
	Q1	589/25323 (2.33)	Ref		< 0.001
	Q2	716/25323 (2.83)	1.19 (1.06–1.33)	0.002	
	Q3	751/25323 (2.97)	1.20 (1.07–1.34)	0.002	
	Q4	1037/25323 (4.10)	1.64 (1.46–1.83)	< 0.001	
Model3	Per 1 SD	3093/101292 (3.05)	1.42 (1.28–1.58)	< 0.001	
	Q1	589/25323 (2.33)	Ref		< 0.001
	Q2	716/25323 (2.83)	1.15 (1.03–1.29)	0.012	
	Q3	751/25323 (2.97)	1.12 (1.00–1.25)	0.057	
	Q4	1037/25323 (4.10)	1.41 (1.25–1.58)	< 0.001	
**ISS**					
Model1	Per 1 SD	2591/101292 (2.56)	1.99 (1.80–2.21)	< 0.001	
	Q1	482/25323 (1.90)	Ref		< 0.001
	Q2	575/25323 (2.27)	1.20 (1.06–1.36)	0.003	
	Q3	637/25323 (2.52)	1.34 (1.19–1.51)	< 0.001	
	Q4	897/25323 (3.54)	1.95 (1.75–2.18)	< 0.001	
Model2	Per 1 SD	2591/101292 (2.56)	1.75 (1.56–1.97)	< 0.001	
	Q1	482/25323 (1.90)	Ref		< 0.001
	Q2	575/25323 (2.27)	1.15 (1.02–1.30)	0.022	
	Q3	637/25323 (2.52)	1.22 (1.08–1.38)	0.002	
	Q4	897/25323 (3.54)	1.68 (1.49–1.90)	< 0.001	

Model3	Per 1 SD	2591/101292 (2.56)	1.49 (1.32–1.67)	< 0.001	
	Q1	482/25323 (1.90)	Ref		< 0.001
	Q2	575/25323 (2.27)	1.12 (0.99–1.26)	0.076	
	Q3	637/25323 (2.52)	1.13 (1.00–1.29)	0.050	
	Q4	897/25323 (3.54)	1.43 (1.26–1.63)	< 0.001	
**Hemorrhagic stroke**					
Model1	Per 1 SD	651/101292 (0.64)	1.15 (1.06–1.24)	0.001	
	Q1	139/25323 (0.55)	Ref		0.002
	Q2	168/25323 (0.66)	1.33 (1.20–1.47)	< 0.001	
	Q3	146/25323 (0.58)	1.82 (1.65–2.00)	< 0.001	
	Q4	198/25323 (0.78)	3.44 (3.16–3.76)	< 0.001	
Model2	Per 1 SD	651/101292 (0.64)	1.17 (1.07–1.28)	< 0.001	
	Q1	139/25323 (0.55)	Ref		0.003
	Q2	168/25323 (0.66)	1.23 (0.98–1.55)	0.069	
	Q3	146/25323 (0.58)	1.09 (0.86–1.39)	0.477	
	Q4	198/25323 (0.78)	1.52 (1.19–1.94)	0.001	
Model3	Per 1 SD	651/101292 (0.64)	1.11 (1.01–1.22)	0.027	
	Q1	139/25323 (0.55)	Ref		0.075
	Q2	168/25323 (0.66)	1.22 (1.16–1.24)	0.088	
	Q3	146/25323 (0.58)	1.06 (0.97–1.52)	0.605	
	Q4	198/25323 (0.78)	1.48 (1.19–1.83)	< 0.001	
**AF**					
Model1	Per 1 SD	8999/101292 (8.88)	2.92 (2.76–3.10)	< 0.001	
	Q1	1370/25323 (5.41)	Ref		< 0.001
	Q2	1780/25323 (7.03)	1.31 (1.22–1.41)	< 0.001	
	Q3	2254/25323 (8.90)	1.68 (1.57–1.79)	< 0.001	
	Q4	3595/25323 (14.20)	2.76 (2.59–2.94)	< 0.001	
Model2	Per 1 SD	8999/101292 (8.88)	2.54 (2.38–v2.71)	< 0.001	
	Q1	1370/25323 (5.41)	Ref		< 0.001
	Q2	1780/25323 (7.03)	1.26 (1.17–1.35)	< 0.001	
	Q3	2254/25323 (8.90)	1.52 (1.42–1.63)	< 0.001	
	Q4	3595/25323 (14.20)	2.35 (2.19–2.52)	< 0.001	
Model3	Per 1 SD	8999/101292 (8.88)	2.18 (2.05-2.33)	< 0.001	
	Q1	1370/25323 (5.41)	Ref		< 0.001
	Q2	1780/25323 (7.03)	1.21 (1.13–1.30)	< 0.001	
	Q3	2254/25323 (8.90)	1.41 (1.31–1.51)	< 0.001	
	Q4	3595/25323 (14.20)	2.03 (1.89–2.18)	< 0.001	
**HF**					
Model1	Per 1 SD	4790/101292 (4.73)	3.98 (3.67–4.32)	< 0.001	
	Q1	655/25323 (2.59)	Ref		< 0.001
	Q2	860/25323 (3.40)	1.33 (1.20–1.47)	< 0.001	

	Q3	1167/25323 (4.61)	1.82 (1.65–2.00)	< 0.001	
	Q4	2108/25323 (8.32)	3.44 (3.16–3.76)	< 0.001	
Model2	Per 1 SD	4790/101292 (4.73)	3.91 (3.56–4.29)	< 0.001	
	Q1	655/25323 (2.59)	Ref		< 0.001
	Q2	860/25323 (3.40)	1.30 (1.18–1.44)	< 0.001	
	Q3	1167/25323 (4.61)	1.77 (1.60–1.95)	< 0.001	
	Q4	2108/25323 (8.32)	3.26 (2.96–3.59)	< 0.001	
Model3	Per 1 SD	4790/101292 (4.73)	2.80 (2.55–3.08)	< 0.001	
	Q1	655/25323 (2.59)	Ref		< 0.001
	Q2	860/25323 (3.40)	1.21 (1.10–1.34)	< 0.001	
	Q3	1167/25323 (4.61)	1.52 (1.37–1.68)	< 0.001	
	Q4	2108/25323 (8.32)	2.45 (2.22–2.70)	< 0.001	

Per increased in 1-SD of METS-VF was 0.436 for UK Biobank

Model 1: Unadjusted

Model 2: Adjusted for age, gender, race, education, smoking, alcohol consumption, and physical activity

Model 3: included all variables from Model 2, and further adjusted for hypertention, diabetes, LDL, Ua and HbA1c.

Abbreviations: METS-VF: Metabolic score for visceral fat; HR:Hazard ratios; CI: Confidence interval; Ref: Reference; SD: Standard Deviation; IHD: Ischemic heart disease; MI: Myocardial infarction; ISS: Ischemic stroke; AF: Atrial fibrillation; HF: Heart failure; LDL-C: Low-density lipoprotein cholesterol; Ua: Uric acid.

Table S5 Associations of METS-VF with heart disease and stroke in CHARLS

Variable	Characteristics	Cases/Total(%)	*HR* (95%CI)	*P value*	*P for trend*
**Heart disease**					
Model1	Per 1 SD	361/1680 (21.49)	3.80 (2.49–5.82)	< 0.001	
	Q1	65/420 (15.48)	Ref		< 0.001
	Q2	70/420 (16.67)	1.20 (0.80–1.79)	0.388	
	Q3	101/420 (24.05)	1.82 (1.25–2.65)	0.002	
	Q4	125/420 (29.76)	2.35 (1.64–3.38)	< 0.001	
Model2	Per 1 SD	361/1680 (21.49)	3.52 (2.49–5.82)	< 0.001	
	Q1	65/420 (15.48)	Ref		< 0.001
	Q2	70/420 (16.67)	1.18 (0.79–1.78)	0.417	
	Q3	101/420 (24.05)	1.86 (1.27–2.72)	0.001	
	Q4	125/420 (29.76)	2.52 (1.70–3.73)	< 0.001	
Model3	Per 1 SD	361/1680 (21.49)	2.51 (1.61–3.78)	< 0.001	
	Q1	65/420 (15.48)	Ref		< 0.001
	Q2	70/420 (16.67)	1.21 (0.82–1.83)	0.361	
	Q3	101/420 (24.05)	1.91 (1.30–2.80)	0.001	
	Q4	125/420 (29.76)	2.58 (1.73–2.84)	< 0.001	
**Stroke**					
Model1	Per 1 SD	159/1680 (9.46)	2.25 (1.41–3.60)	0.001	
	Q1	30/420 (7.14)	Ref		< 0.001
	Q2	35/420 (8.33)	0.84 (0.52–1.38)	0.508	
	Q3	35/420 (8.33)	0.99 (0.62–1.59)	0.975	
	Q4	59/420 (14.05)	1.73 (1.14–2.63)	0.01	
Model2	Per 1 SD	159/1680 (9.46)	1.72 (1.05–2.81)	0.031	
	Q1	30/420 (7.14)	Ref		< 0.001
	Q2	35/420 (8.33)	0.84 (0.52–1.38)	0.494	
	Q3	35/420 (8.33)	0.99 (0.62–1.58)	0.954	
	Q4	59/420 (14.05)	1.67 (1.08–2.57)	0.02	
Model3	Per 1 SD	159/1680 (9.46)	1.82 (1.11–3.01)	0.018	
	Q1	30/420 (7.14)	Ref		< 0.001
	Q2	35/420 (8.33)	0.88 (0.54–1.43)	0.596	
	Q3	35/420 (8.33)	0.99 (0.62–1.59)	0.964	
	Q4	59/420 (14.05)	1.63 (1.05–2.53)	0.031	

Per increased in 1-SD of METS-VF was 0.384 for CHARLS

Model 1: Unadjusted

Model 2: Adjusted for age, gender, education, smoking, and alcohol consumption

Model 3: included all variables from Model 2, and further adjusted for hypertention, diabetes, LDL, Ua and HbA1c.

Abbreviations: METS-VF: Metabolic score for visceral fat; HR: Hazard ratios; CI: Confidence interval; Ref: Reference; SD: Standard Deviation; LDL-C: Low-density lipoprotein cholesterol; Ua: Uric acid.

Table. S6 Threshold effect analysis of METS-VF for new-onset CVD in UKB

Variables	Adjusted *HR (95% CI), P value*
**CVD**	
Model1 Standard Line regression	1.64 (1.58–1.71) , $P < 0.001$
Model2 Two-piece wise linear regression	
Inflection point	7.285
METS-VF < 7.285	1.30 (1.23–2.25) , $P < 0.001$
METS-VF ≥ 7.285	2.45 (2.26–2.67) , $P < 0.001$
Log-likelihood ratio test	$P < 0.001$
**IHD**	
Model1 Standard Line regression	1.44 (1.36–1.52) , $P < 0.001$
Model2 Two-piece wise linear regression	
Inflection point	7.224
METS-VF < 7.224	1.19 (1.09–1.29) , $P < 0.001$
METS-VF ≥ 7.224	1.87 (1.69–2.09) , $P < 0.001$
Log-likelihood ratio test	$P < 0.001$
**Angina**	
Model1 Standard Line regression	1.42 (1.32–1.54) , $P < 0.001$
Model2 Two-piece wise linear regression	
Inflection point	7.193
METS-VF < 7.193	1.24 (1.10–1.40) , $P < 0.001$
METS-VF ≥ 7.193	1.71 (1.48–1.97) , $P < 0.001$
Log-likelihood ratio test	$P = 0.003$
**Stroke**	
Model1 Standard Line regression	1.42 (1.28–1.58) , $P < 0.001$
Model2 Two-piece wise linear regression	
Inflection point	7.213
METS-VF < 7.213	1.21 (1.04–1.42) , $P = 0.015$
METS-VF ≥ 7.213	1.79 (1.46–2.19) , $P < 0.001$
Log-likelihood ratio test	$P = 0.009$

**ISS**	
Model1 Standard Line regression	1.42 (1.32–1.67) , $P < 0.001$
Model2 Two-piece wise linear regression	
Inflection point	7.225
METS-VF < 7.225	1.24 (1.05–1.48) , $P = 0.012$
METS-VF ≥ 7.225	1.91 (1.54–2.38) , $P < 0.001$
Log-likelihood ratio test	$P = 0.009$
**Hemorrhagic stroke**	
Model1 Standard Line regression	1.21 (0.97–1.51) , $P = 0.088$
Model2 Two-piece wise linear regression	
Inflection point	6.928
METS-VF < 6.928	0.85 (0.57–1.26) , $P = 0.413$
METS-VF ≥ 6.928	1.56 (1.12–2.17) , $P = 0.009$
Log-likelihood ratio test	$P = 0.049$
**AF**	
Model1 Standard Line regression	2.18 (2.04–2.33) , $P < 0.001$
Model2 Two-piece wise linear regression	
Inflection point	7.361
METS-VF < 7.361	1.60 (1.46–1.74) , $P < 0.001$
METS-VF ≥ 7.361	3.99 (3.48–4.58) , $P < 0.001$
Log-likelihood ratio test	$P < 0.001$
**HF**	
Model1 Standard Line regression	2.80 (2.55–3.08) , $P < 0.001$
Model2 Two-piece wise linear regression	
Inflection point	7.440
METS-VF < 7.440	1.89 (1.68–2.13) , $P < 0.001$
METS-VF ≥ 7.440	6.99 (5.69–8.57) , $P < 0.001$
Log-likelihood ratio test	$P < 0.001$

Abbreviations: METS-VF: Metabolic score for visceral fat; HR: Hazard ratios; CI: Confidence interval; Ref: Reference; CVD: Cardiovascular disease; HR: Hazard ratios; CI: Confidence interval. IHD: Ischemic heart disease; MI: Myocardial infarction; ISS: Ischemic stroke; AF:

Atrial fibrillation; HF: Heart failure

Table S7 Threshold effect analysis of METS-VF for new-onset CVD in CHARLS

**Variables**	**Adjusted** ***HR (95% CI), P value***
**Stroke**	
Model1 Standard Line regression	2.11 (1.29–3.46) , *P* = 0.002
Model2 Two-piece wise linear regression	
Inflection point	7.068
METS-VF < 7.068	1.14 (0.63–2.07) , *P* = 0.653
METS-VF ≥ 7.068	5.54 (2.51–12.22) , *P* < 0.001
Log-likelihood ratio test	*P* = 0.009

Abbreviations: METS-VF: Metabolic score for visceral fat; CVD: Cardiovascular disease; HR: Hazard ratios; CI: Confidence interval. IHD: Ischemic heart disease; MI: Myocardial infarction; ISS: Ischemic stroke; AF: Atrial fibrillation; HF: Heart failure

Table S8 Joint association of METS-VF and PRS with new-onset CVD in UKB

**Variables**	**Genetic risk**	**METS-VF**	***HR (95%CI)***	***P value***
CVD	Low	Q1	Ref	
	Low	Q2	1.10 (0.98–1.23)	0.108
	Low	Q3	1.25 (1.12–1.39)	< 0.001
	Low	Q4	1.78 (1.61–1.97)	< 0.001
	Intermediate	Q1	1.20 (1.09–1.32)	< 0.001
	Intermediate	Q2	1.37 (1.25–1.51)	< 0.001
	Intermediate	Q3	1.58 (1.44–1.73)	< 0.001
	Intermediate	Q4	1.99 (1.82–2.18)	< 0.001
	High	Q1	1.52 (1.37–1.69)	< 0.001
	High	Q2	1.87 (1.69–.07)	< 0.001
	High	Q3	1.89 (1.70–2.09)	< 0.001
	High	Q4	2.36 (2.14–2.61)	< 0.001
IHD	Low	Q1	Ref	
	Low	Q2	1.00 (0.84–1.19)	0.979
	Low	Q3	1.13 (0.96–1.34)	0.142
	Low	Q4	1.62 (1.39–1.89)	< 0.001
	Intermediate	Q1	1.40 (1.21–1.62)	< 0.001
	Intermediate	Q2	1.60 (1.39–2.06)	< 0.001
	Intermediate	Q3	1.79 (1.55–2.61)	< 0.001
	Intermediate	Q4	2.16 (1.88–2.48)	< 0.001
	High	Q1	2.32 (1.99–2.70)	< 0.001
	High	Q2	2.56 (2.20–2.97)	< 0.001
	High	Q3	2.66 (2.29–3.09)	< 0.001
	High	Q4	3.05 (2.63–3.53)	< 0.001
Angina	Low	Q1	Ref	
	Low	Q2	1.07 (0.84–1.36)	0.574
	Low	Q3	1.11 (0.88–1.39)	0.389
	Low	Q4	1.42 (1.14–1.77)	0.002
	Intermediate	Q1	1.40 (1.16–1.70)	0.001
	Intermediate	Q2	1.56 (1.29–1.89)	< 0.001
	Intermediate	Q3	1.85 (1.53–2.24)	< 0.001
	Intermediate	Q4	2.14 (1.77–2.59)	< 0.001
	High	Q1	2.26 (1.84–2.78)	< 0.001
	High	Q2	2.60 (2.13–3.19)	< 0.001
	High	Q3	2.71 (2.21–3.31)	< 0.001
	High	Q4	3.04 (2.49–3.72)	< 0.001
MI	Low	Q1	Ref	
	Low	Q2	1.31 (0.95–1.81)	0.097
	Low	Q3	1.31 (0.96–1.79)	0.094
	Low	Q4	1.62 (1.20–2.19)	0.002

	Intermediate	Q1	1.76 (1.35–2.30)	< 0.001
	Intermediate	Q2	2.03 (1.56–2.65)	< 0.001
	Intermediate	Q3	2.52 (1.94–3.27)	< 0.001
	Intermediate	Q4	3.20 (2.47–4.15)	< 0.001
	High	Q1	3.06 (2.32–4.04)	< 0.001
	High	Q2	3.73 (2.84–4.90)	< 0.001
	High	Q3	4.32 (3.29–5.67)	< 0.001
	High	Q4	4.43 (3.38–5.82)	< 0.001
ISS	Low	Q1	Ref	
	Low	Q2	1.04 (0.75–1.43)	
	Low	Q3	0.92 (0.66–1.27)	0.832
	Low	Q4	1.53 (1.14–2.05)	0.611
	Intermediate	Q1	1.25 (0.96–1.63)	0.005
	Intermediate	Q2	1.43 (1.11–1.85)	0.096
	Intermediate	Q3	1.46 (1.13–1.89)	0.007
	Intermediate	Q4	1.78 (1.38–2.31)	0.004
	High	Q1	1.77 (1.32–2.36)	< 0.001
	High	Q2	1.90 (1.43–2.52)	< 0.001
	High	Q3	2.07 (1.56–2.73)	< 0.001
	High	Q4	2.34 (1.77–3.07)	< 0.001
AF	Low	Q1	Ref	
	Low	Q2	1.27 (1.01–1.59)	0.039
	Low	Q3	1.41 (1.14–1.75)	0.002
	Low	Q4	2.34 (1.92–2.85)	< 0.001
	Intermediate	Q1	1.88 (1.56–2.26)	< 0.001
	Intermediate	Q2	2.27 (1.89–2.72)	< 0.001
	Intermediate	Q3	2.58 (2.15–3.09)	< 0.001
	Intermediate	Q4	3.81 (3.18–4.56)	< 0.001
	High	Q1	3.41 (2.81–4.14)	< 0.001
	High	Q2	4.16 (3.44–5.03)	< 0.001
	High	Q3	5.06 (4.20–6.09)	< 0.001
	High	Q4	6.72 (5.59–8.07)	< 0.001

Adjusted for age, gender, race, education, smoking, alcohol consumption, physical activity, hypertention, diabetes, LDL-C, Ua and HbA1c. Abbreviations: METS-VF: Metabolic score for visceral fat; CVD: Cardiovascular disease; PRS: Polygenic risk score; HR: Hazard ratios; CI: Confidence interval; Ref: Reference; IHD: Ischemic heart disease; MI: Myocardial infarction; ISS: Ischemic stroke; AF: Atrial fibrillation; LDL-C: Low-density lipoprotein cholesterol; Ua: Uric acid

Table S9 The predictive ability of METS-VF for the risk of new-onset CVD

Variables	NRI (95% CI)	*P value*	IDI (95% CI)	*P value*
UK biobank				
METS-VF VS METS-IR（3 years）	0.165 (0.075–0.177)	＜ 0.001	0.003 (0.001–0.004)	＜ 0.001
METS-VF VS BMI（3 years）	0.164 (0.041–0.178)	＜ 0.001	0.004 (0.001–0.004)	＜ 0.001
METS-VF VS TyG（3 years）	0.137 (0.033–0.152)	＜ 0.001	0.004 (0.001–0.004)	＜ 0.001
METS-VF VS VAI（3 years）	0.138 (0.007–0.148)	＜ 0.001	0.004 (0–0.004)	＜ 0.001
METS-VF VS METS-IR（5 years）	0.170 (0.041–0.180)	＜ 0.001	0.006 (0.001–0.007)	＜ 0.001
METS-VF VS BMI（5 years）	0.167 (0.004–0.176)	＜ 0.001	0.007 (0.002–0.007)	＜ 0.001
METS-VF VS TyG（5 years）	0.142 (0.034–0.154)	＜ 0.001	0.007 (0.002–0.008)	＜ 0.001
METS-VF VS VAI（5 years）	0.140 (0.007–0.140)	＜ 0.001	0.007 (0–0.007)	＜ 0.001
METS-VF VS METS-IR（8 years）	0.168 (0.041–0.174)	＜ 0.001	0.011 (0.003–0.011)	＜ 0.001
METS-VF VS BMI（8 years）	0.165 (0.041–0.168)	＜ 0.001	0.011 (0.003–0.012)	＜ 0.001
METS-VF VS TyG（8 years）	0.142 (0.035–0.146)	＜ 0.001	0.012 (0.003–0.013)	＜ 0.001
METS-VF VS VAI（8 years）	0.141 (0.007–0.142)	＜ 0.001	0.012 (0.001–0.012)	＜ 0.001
CHARLS				
METS-VF VS METS-IR（3 years）	0.173 (0.013–0.335)	＜ 0.001	0.006 (0.001–0.011)	＜ 0.001
METS-VF VS BMI（3 years）	0.174 (0.013–0.335)	＜ 0.001	0.006 (0.001–0.011)	＜ 0.001
METS-VF VS TyG（3 years）	0.257 (0.017–0.339)	＜ 0.001	0.006 (0–0.01)	＜ 0.001
METS-VF VS VAI（3 years）	0.174 (0.009–0.289)	＜ 0.001	0.005 (0–0.008)	＜ 0.001
METS-VF VS METS-IR（5 years）	0.150(0.019–0.258)	＜ 0.001	0.005 (0–0.011)	＜ 0.001
METS-VF VS BMI（5 years）	0.150(0.019–0.258)	＜ 0.001	0.005 (0–0.011)	＜ 0.001
METS-VF VS TyG（5 years）	0.169 (0.025–0.225)	＜ 0.001	0.005 (0–0.01)	＜ 0.001
METS-VF VS VAI（5 years）	0.125 (0.008–0.213)	＜ 0.001	0.004 (0–0.008)	＜ 0.001
METS-VF VS METS-IR（8 years）	0.147 (0.034–0.199)	＜ 0.001	0.022 (0.004–0.03)	＜ 0.001
METS-VF VS BMI（8 years）	0.147 (0.034–0.199)	＜ 0.001	0.022 (0.004–0.03)	＜ 0.001
METS-VF VS TyG（8 years）	0.163 (0.028–0.172)	＜ 0.001	0.022 (0.003–0.029)	＜ 0.001
METS-VF VS VAI（8 years）	0.146 (0.001–0.158)	＜ 0.001	0.02 (0.01–0.022)	＜ 0.001

Abbreviations: CI: Confidence interval; IDI: Integrated discrimination improvement; NRI: Net reclassification improvement; CVD: Cardiovascular disease; METS-VF: Metabolic score for visceral fat; METS-IR: Metabolic score for insulin resistance; BMI: Body mass index (BMI); TyG: Triglyceride-glucose index; VAI: Visceral adiposity index.

Table S10. Competing risk analysis of the association between METS-VF and new-onset CVD

Variables	Characteristic	Cases/Total (%)	sHR (95% CI)	P value	P for trend
**UK Biobank**	Per 1 SD	21231/101292 (20.96)	1.58 (1.51–1.66)	<0.001	–
	Q1	3709/25323 (14.65)	Ref	–	< 0.001
	Q2	4535/25323 (17.91)	1.12 (1.07–1.17)	<0.001	
	Q3	5442/25323 (21.49)	1.24 (1.18–1.29)	<0.001	
	Q4	7545/25323 (29.80)	1.57 (1.50–1.64)	<0.001	
**CHARLS**	Per 1 SD	361/1680 (21.49)	1.34 (1.22–1.48)	<0.001	–
	Q1	65/420 (15.48)	Ref	–	< 0.001
	Q2	70/420 (16.67)	1.08 (0.77–1.52)	0.672	
	Q3	101/420 (24.05)	1.59 (1.15–2.18)	0.011	
	Q4	125/420 (29.76)	2.04 (1.47–2.83)	<0.001	

Adjusted for age, gender, race (UK Biobank only), education, smoking, alcohol consumption, physical activity (UK Biobank only), hypertention, diabetes, LDL-C, Ua and HbA1c. Abbreviations: METS-VF: Metabolic score for visceral fat; CVD: Cardiovascular disease; SD: Standard Deviation; HR: Hazard ratios; CI: Confidence interval; Ref: Reference; LDL-C: Low-density lipoprotein cholesterol; Ua:Uric acid.

Table S11. Sensitivity analyses of the association between METS-VF and new-onset CVD excluding metabolic variables

Variables	Characteristic	Cases/Total (%)	*HR (95% CI)*	*P value*	*P for trend*
**UK Biobank**	Per 1 SD	21231/101292 (20.96)	1.21 (1.17–1.28)	< 0.001	–
	Q1	3709/25323 (14.65)	Ref	–	< 0.001
	Q2	4535/25323 (17.91)	1.22 (1.17–1.28)	< 0.001	
	Q3	5442/25323 (21.49)	1.39 (1.33–1.44)	< 0.001	
	Q4	7545/25323 (29.80)	1.82 (1.75–1.90)	< 0.001	
**CHARLS**	Per 1 SD	361/1680 (21.49)	1.52 (1.38–1.69)	< 0.001	–
	Q1	65/420 (15.48)	Ref	–	< 0.001
	Q2	70/420 (16.67)	1.18 (0.84–1.67)	0.345	
	Q3	101/420 (24.05)	1.79 (1.29–2.47)	< 0.001	
	Q4	125/420 (29.76)	2.36 (1.70–3.28)	< 0.001	

Adjusted for age, gender, race (UK Biobank only), education, smoking, alcohol consumption, physical activity (UK Biobank only), and Ua. Abbreviations: METS-VF: Metabolic score for visceral fat; CVD: Cardiovascular disease; HR: Hazard ratios; CI: Confidence interval; Ref: Reference; SD: Standard Deviation; Ua:Uric acid.

Table S12. Sensitivity analyses of the association between METS-VF and new-onset CVD excluding participants taking medications

Variables	Characteristic	Cases/Total (%)	*HR (95% CI)*	*P value*	*P for trend*
**UK Biobank**	Per 1 SD	7186/47006 (15.29)	1.78 (1.65–1.92)	< 0.001	–
	Q1	1405/13249 (10.60)	Ref	–	< 0.001
	Q2	1681/12442 (13.51)	1.21 (1.12–1.23)	< 0.001	
	Q3	1962/11716 (16.75)	1.38 (1.28–1.49)	< 0.001	
	Q4	2138/9599 (22.27)	1.73 (1.60–1.87)	< 0.001	
**CHARLS**	Per 1 SD	169/930 (18.17)	1.35 (1.16–1.57)	< 0.001	–
	Q1	38/275 (13.82)	Ref	–	< 0.001
	Q2	35/245 (14.29)	1.04 (0.65–1.67)	0.859	
	Q3	50/235 (21.28)	1.60 (1.04–2.47)	0.033	
	Q4	46/175 (26.29)	1.85 (1.23–2.85)	0.001	

Adjusted for age, gender, race (UK Biobank only), education, smoking, alcohol consumption, physical activity (UK Biobank only), hypertention, diabetes, LDL-C, Ua and HbA1c. Abbreviations: METS-VF: Metabolic score for visceral fat; CVD: Cardiovascular disease; SD: Standard Deviation; HR: Hazard ratios; CI: Confidence interval; Ref: Reference; LDL-C: Low-density lipoprotein cholesterol; Ua:Uric acid.

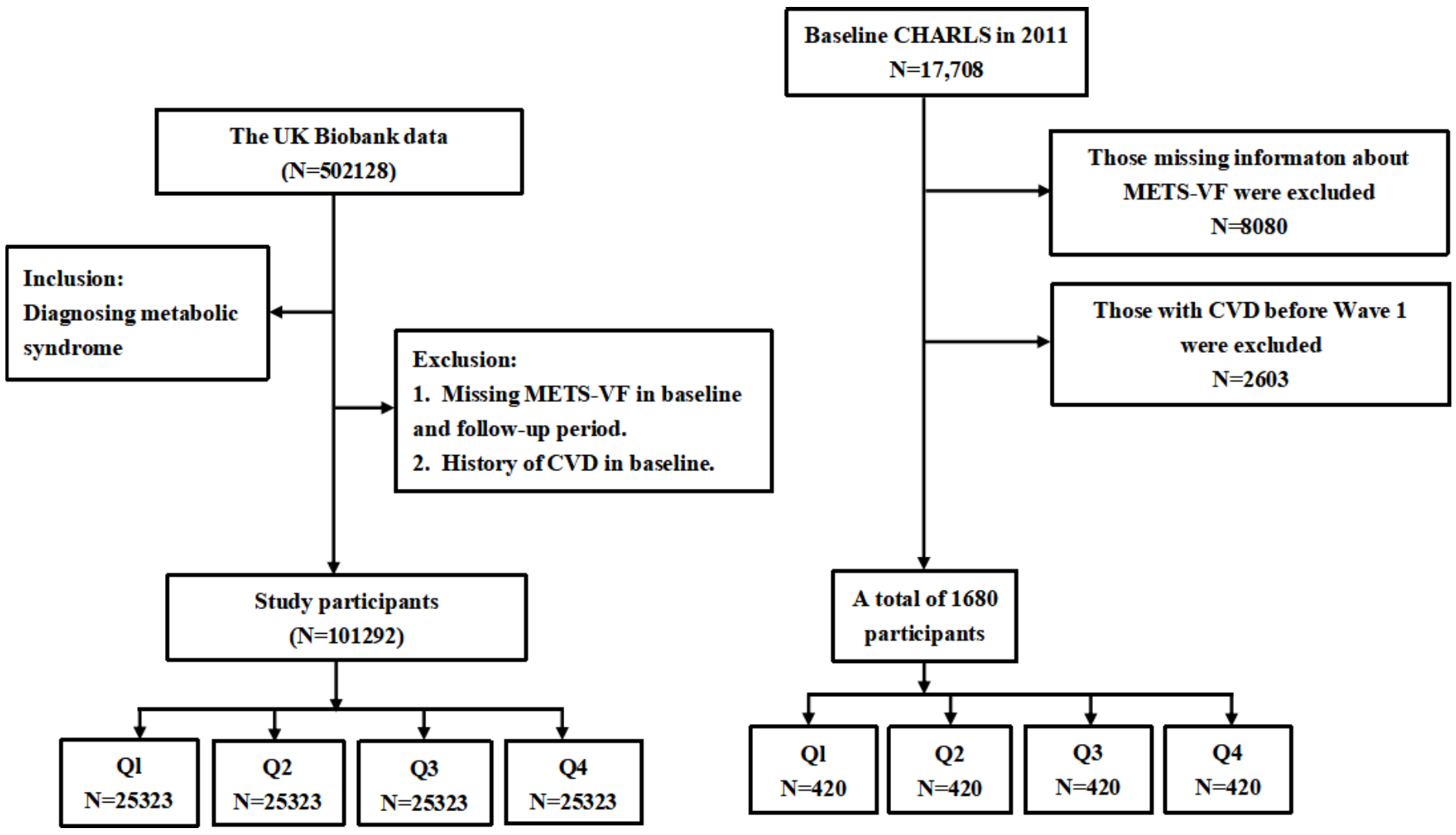

Fig. S1 Study workflow in UK Biobank and CHARLS

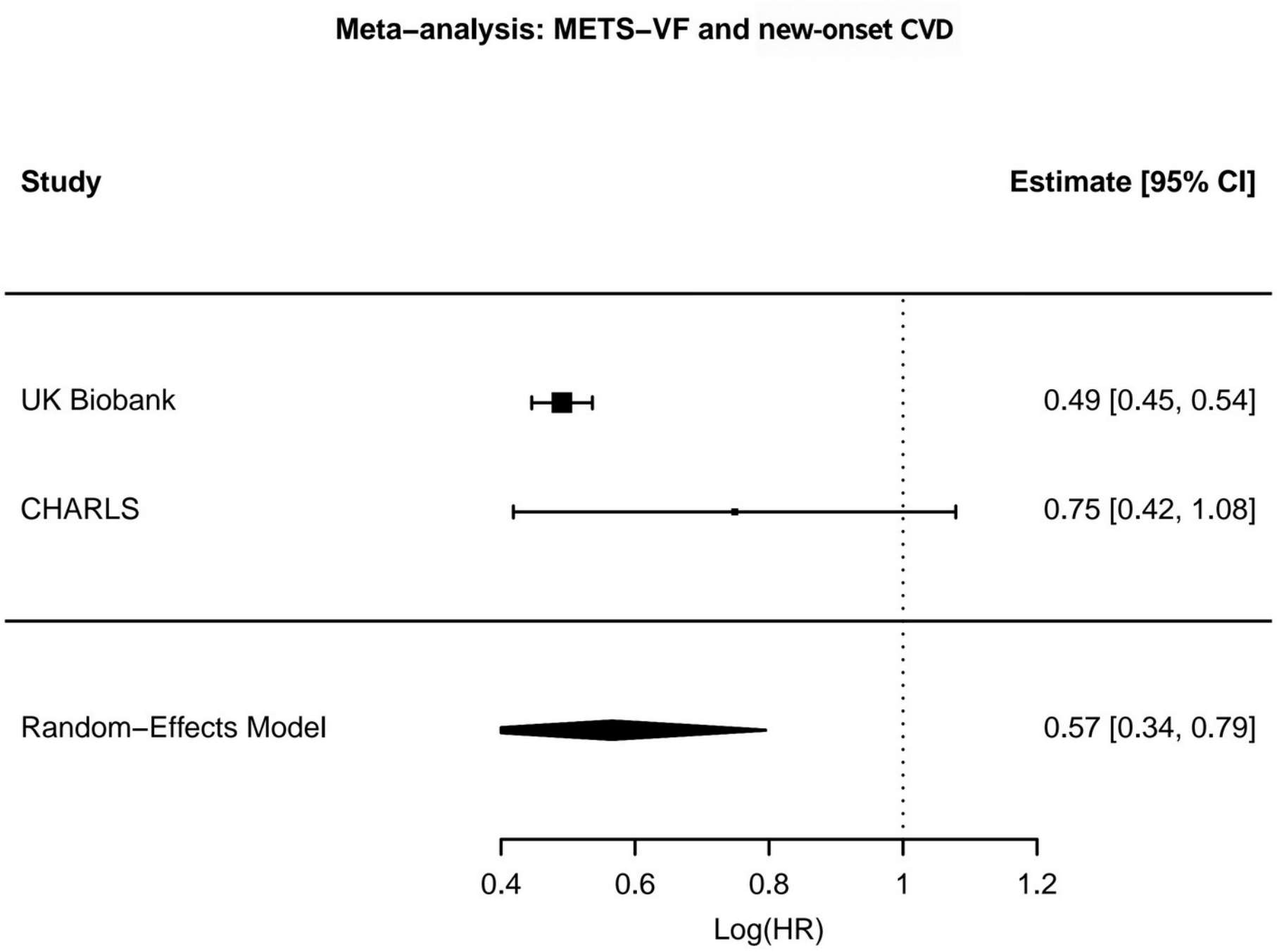

Cardiovascular disease.

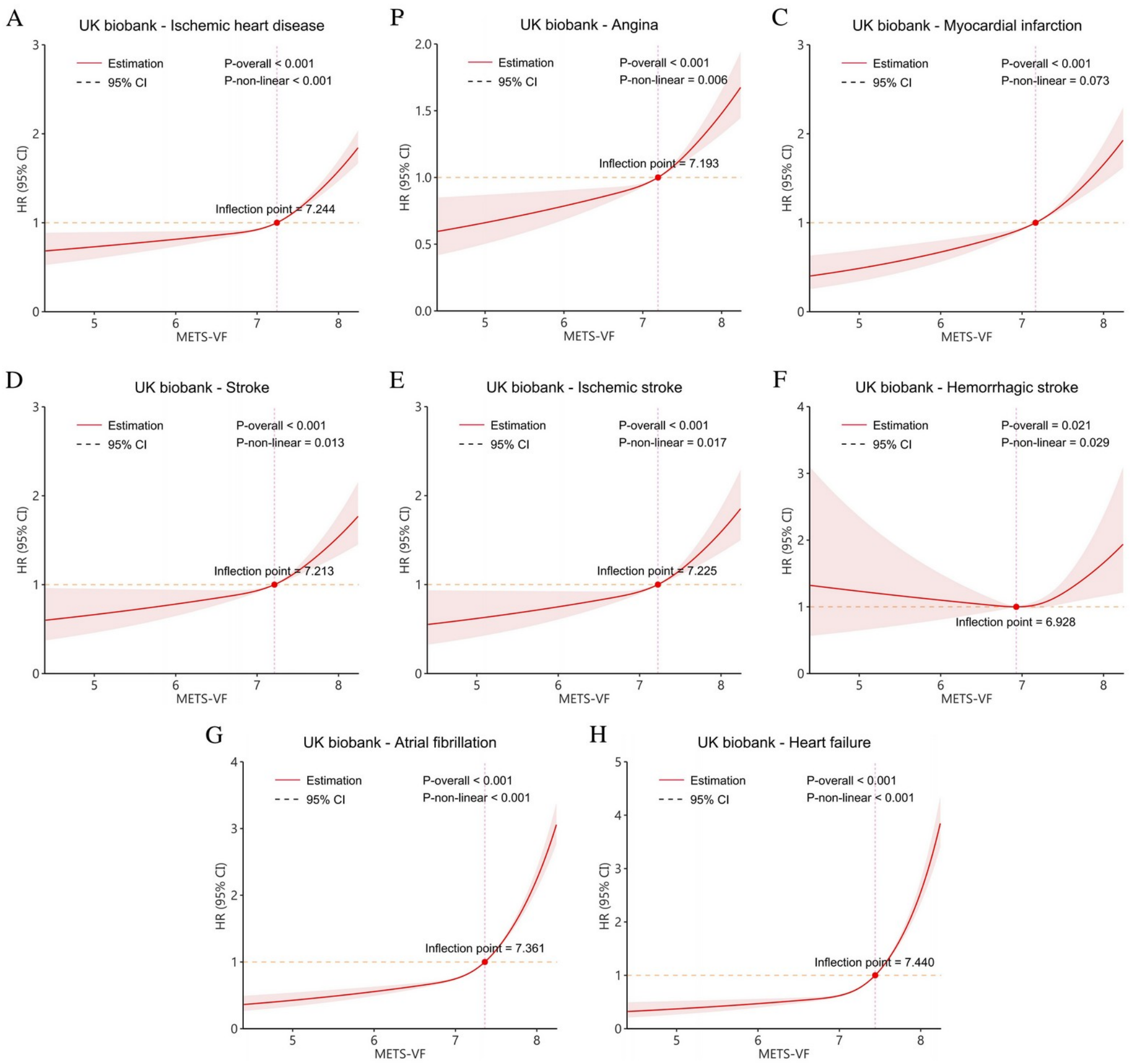

Fig. S3 Analysis of restricted cubic spline regression in UK biobank. (A) Non-linear relationship between the METS-VF with IHD. (B) Non-linear relationship between the METS-VF with angina. (C) Positive linear relationship between the METS-VF with MI. (D) (F) Non-linear relationship between the METS-VF with stroke. (E) Non-linear relationship between the METS-VF with ISS (F) Non-linear relationship between the METS-VF with Hemorrhagic stroke. (G) Non-linear relationship between the METS-VF with AF. (H) Non-linear relationship between the METS-VF with HF. Adjusted for age, gender, race, education level, smoking status, alcohol consumption status, physical activity, hypertention, diabetes, LDL-C, Ua and HbA1c. Abbreviations: HR: Hazard ratios; CI: Confidence interval; METS-VF: Metabolic score for visceral fat; LDL-C: Low-density lipoprotein cholesterol; Ua: Uric acid; MI: Myocardial infarction; ISS: Ischemic stroke; AF: Atrial fibrillation; HF: Heart failure.

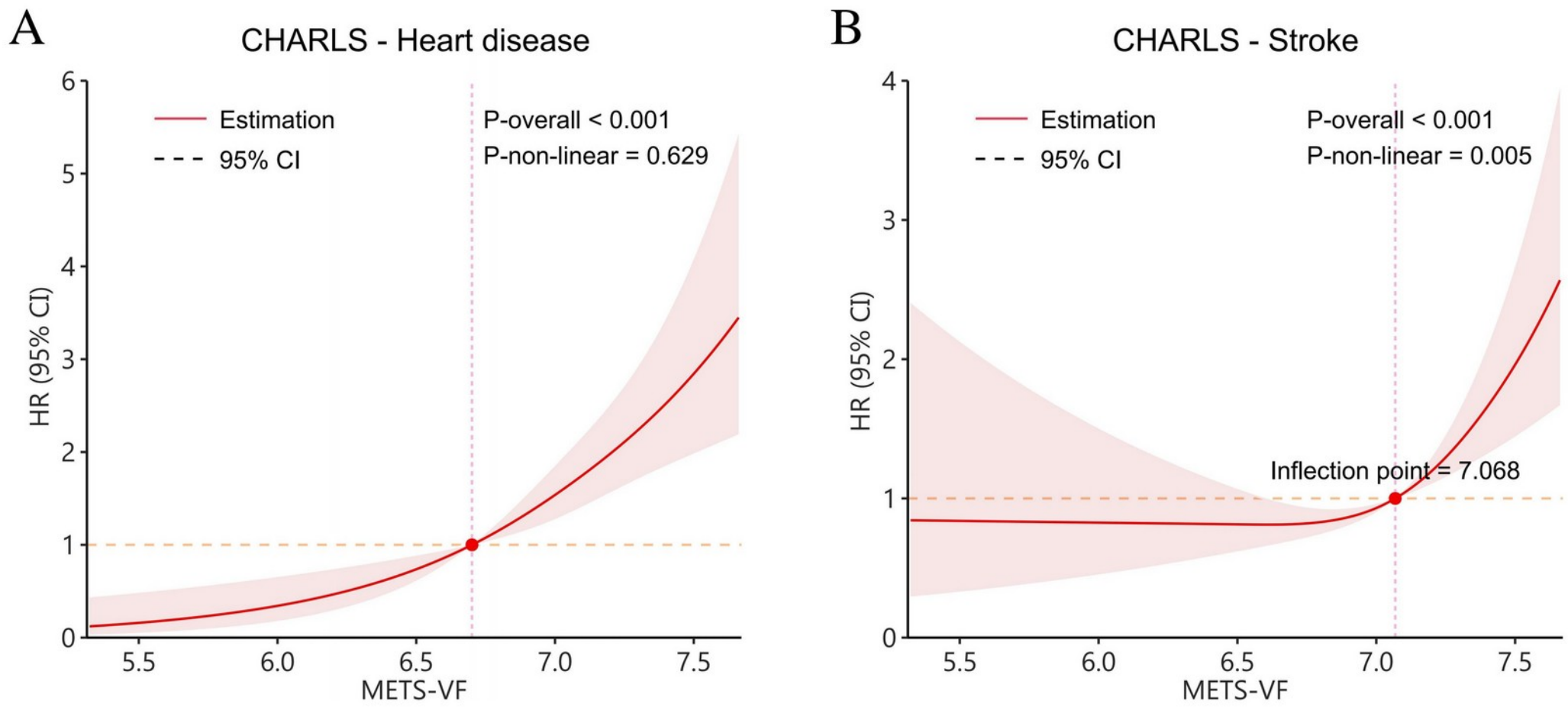

Fig. S4 Analysis of restricted cubic spline regression in CHARLS. (A) Positive linear relationship between the METS-VF with Heart disease. (B) Non-linear relationship between the METS-VF with stroke. Adjusted for age, gender, educationl, smoking, alcohol consumption, hypertention, diabetes, LDL-C, Ua and HbA1c. Abbreviations: HR: Hazard ratios; CI: Confidence interval; METS-VF: Metabolic score for visceral fat; AF: Atrial fibrillation; LDL-C: Low-density lipoprotein cholesterol; Ua: Uric acid.
